# Quantitative risk assessment of haemolytic uremic syndrome associated with beef consumption in Argentina

**DOI:** 10.1371/journal.pone.0242317

**Published:** 2020-11-13

**Authors:** Victoria Brusa, Magdalena Costa, Nora L. Padola, Analía Etcheverría, Fernando Sampedro, Pablo S. Fernandez, Gerardo A. Leotta, Marcelo L. Signorini

**Affiliations:** 1 IGEVET–Instituto de Genética Veterinaria “Ing. Fernando N. Dulout” (UNLP-CONICET LA PLATA), Facultad de Ciencias Veterinarias UNLP, Buenos Aires, Argentina; 2 CIVETAN–Centro de Investigación Veterinaria de Tandil (CONICET-UNCPBA-CICPBA), Facultad de Ciencias Veterinarias—UNCPBA, Buenos Aires, Argentina; 3 Environmental Health Sciences Division, School of Public Health, University of Minnesota, Minneapolis, United States of America; 4 Escuela Técnica Superior de Ingeniería Agronómica, Universidad Politécnica de Cartagena, España; 5 IdICaL–Instituto de Investigación de la Cadena Láctea–(INTA–CONICET), Santa Fe, Argentina; University of Campinas, BRAZIL

## Abstract

We developed a quantitative microbiological risk assessment (QMRA) of haemolytic uremic syndrome (HUS) associated with Shiga toxin-producing *Escherichia coli* (STEC)-contaminated beef (intact beef cuts, ground beef and commercial hamburgers) in children under 15 years of age from Argentina. The QMRA was used to characterize STEC prevalence and concentration levels in each product through the Argentinean beef supply chain, including cattle primary production, cattle transport, processing and storage in the abattoir, retail and home preparation, and consumption. Median HUS probability from beef cut, ground beef and commercial hamburger consumption was <10^−15^, 5.4x10^-8^ and 3.5x10^-8^, respectively. The expected average annual number of HUS cases was 0, 28 and 4, respectively. Risk of infection and HUS probability were sensitive to the type of abattoir, the application or not of Hazard Analysis and Critical Control Points (HACCP) for STEC (HACCP-STEC), *stx* prevalence in carcasses and trimmings, storage conditions from the abattoir to retailers and home, the joint consumption of salads and beef products, and cooking preference. The QMRA results showed that the probability of HUS was higher if beef cuts (1.7x) and ground beef (1.2x) were from carcasses provided by abattoirs not applying HACCP-STEC. Thus, the use of a single sanitary standard that included the application of HACCP-STEC in all Argentinean abattoirs would greatly reduce HUS incidence. The average number of annual HUS cases estimated by the QMRA (n = 32) would explain about 10.0% of cases in children under 15 years per year in Argentina. Since other routes of contamination can be involved, including those not related to food, further research on the beef production chain, other food chains, person-to-person transmission and outbreak studies should be conducted to reduce the impact of HUS on the child population of Argentina.

## 1. Introduction

Shiga toxin-producing *Escherichia coli* (STEC) are foodborne pathogens associated with a wide spectrum of human diseases, from mild diarrhea to hemorrhagic colitis, thrombocytopenia and haemolytic uremic syndrome (HUS), which can lead to death [1]. Information about HUS cases around the world is scarce, particularly primary studies and notifiable disease data from different World Health Organization (WHO) regions, and population estimates on exposure, age distribution and clinical course of illness [2].

An estimated 2.5 million new STEC annual cases from different sources, including foodborne, have been reported globally, which have been responsible for 3,330 HUS cases, 269 deaths and 27,000 disability-adjusted life years [3]. In Argentina, STEC are the primary etiological agent of post-enteric HUS, and serotype O157:H7 is most frequently associated with HUS confirmed cases [4]. Between 2011 and 2015, 1,953 HUS cases were reported in Argentina, 70.7% of which corresponded to *E*. *coli* O157:H7 [5]. However, the food vehicle (ground beef and dry sausage) was identified in only four cases [6]. The last available report confirmed 290 HUS cases in 2019 [7].

Unlike Argentina, notification of HUS cases is not mandatory in most countries [8]. The annual HUS incidence rate in the general population of Argentina (0.6 cases per 100,000 inhabitants) [7] is similar to that reported in Canada (1.9) [9], Uruguay (0.4) (G. Varela, pers comm) and Australia (0.07) [10]). The Argentinean surveillance network has allowed the identification of most HUS cases, either in outbreaks or as sporadic cases [11], reporting one of the highest HUS incidence rates in populations younger than 5 and 1 year (6.3 and 12.9 per 100,000 children, respectively) [7]. In other countries, HUS incidence rates per 100,000 children under 5 years are 5.4 in Uruguay (G. Varela, pers comm), 4.2 in Canada [9] and 1.4 in USA [12]. Despite the high incidence rate, HUS-associated mortality rate in Argentina is higher (1.7%) [6] than that reported in Uruguay (1.2%) [G. Varela, pers comm] and lower than that reported in the USA (2.5%) [12], Chile (2.7%) [13] and Australia (12.0%) [10].

Cattle are the main animal reservoir of STEC currently known [14]. Recent reports have also pointed out the role of asymptomatic carriers in person-to-person STEC transmission (fecal-oral route) [6,15–17]. A study conducted in Argentina also showed that living in a farm or being in contact with farm animals and the presence of children <5 years of age in the family attending daycare or kindergarten were among the highest risk factors for STEC infection [18].

It has been recently shown that around 60.0% of all STEC reported cases worldwide cannot be attributed to a food source [19], despite 40.0% of cases were associated with food, mainly beef (18.2%), vegetables (15.6%) and dairy products (5.5%) [19]. In Argentina, beef per capita consumption is 51.0 kg/person [20]. Beef abattoirs can be classified into two main categories, namely, abattoirs with a Hazard Analysis and Critical Control Point (HACCP) system, that defines STEC as hazardous (hereinafter referred to as “applying HACCP-STEC”), and abattoirs with no HACCP plans or HACCP plans that do not define STEC as a hazard (hereinafter referred to as “not applying HACCP-STEC”) [21]. Abattoirs applying HACCP-STEC (38.0%) include cattle from arrival up to the production of vacuum-packaged beef cuts, commercial hamburgers and ground beef for supermarkets (with health authority permission), all within the abattoir plant. In abattoirs classified as “not applying HACCP-STEC” (62.0%), half carcasses are transported to retailers for cutting and deboning to produce beef cuts and for mincing to produce ground beef. In the case of butcher shops, they do not apply HACCP plans and they exceptionally apply good manufacturing practices (GMP) [22], considering that they should mince ground beef in the presence of the consumer according to the Argentine Food Code [23].

Beef can be cross-contaminated with STEC at different stages of the supply chain, from the abattoir to retail and the home environment [24–27]. In intact beef cuts, contamination is superficial, so that STEC can be easily destroyed by cooking [28]. Ground beef is not only considered a high-risk product due to the contamination spread during production, but it is normally associated with eating undercooked meat [29,30]. Additionally, home-made ground beef and commercial hamburgers have also been associated with STEC cases [31]. The prevalence of STEC in different beef products varies globally, ranging from 1.8–57.6% in Argentina to 0.7–60.6% in the rest of the world (S1 Table).

The use of risk analysis has been accepted internationally as a logical sequence of steps that contributes to the implementation of risk management measures based on scientific evidence. Risk assessment, the scientific process component, is the most relevant tool for assessing the association between existing foodborne hazards and public health risks [32]. Several quantitative microbial risk assessment (QMRA) models have been developed to link the presence of STEC in beef products with the risk of developing HUS in a certain population [25,27,30,33–36]. In 2009, a QMRA was developed in Argentina to model STEC contamination of beef hamburgers, using a farm-to-table risk approach [37]. More recent studies about STEC prevalence and contamination levels have been performed in other beef commodities, including hamburger, ground beef and beef cuts [38–43]. In this context, an updated QMRA including this new information would provide an accurate estimate of the incidence of HUS attributed to beef consumption in different age groups.

The aim of this study was to perform a quantitative risk assessment of HUS associated with the consumption of STEC-contaminated beef (intact beef cuts, ground beef and commercial hamburgers) from two abattoir systems in children under 15 years of age from Argentina.

## 2. Materials and methods

### 2.1. Study design

**Fig 1:**
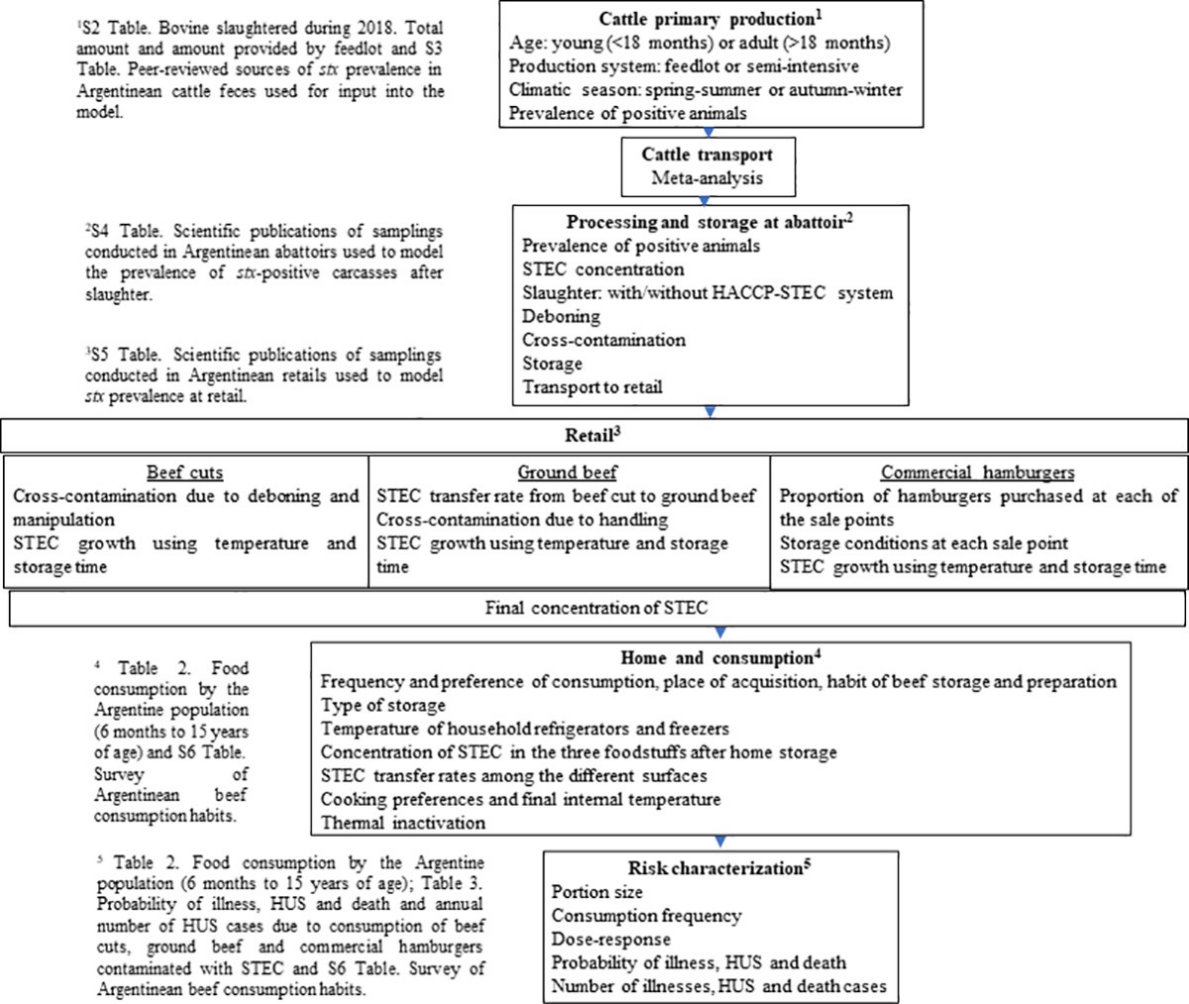
Beef supply chain conceptual model and relevant input variables. ^1^ S2 and S3 Tables, ^2^ S4 Table, ^3^ S5 Table, ^4^ Table 2 and S6 Table, ^5^Tables 2 and 3 and S6 Table.

A probabilistic risk assessment model was developed to characterize STEC prevalence and contamination levels through the beef supply chain (Fig 1). The beef supply chain was divided into five production modules: cattle primary production, cattle transport, processing and storage in the abattoir, retail and home preparation, and consumption. Three beef products were modelled using the production modules described in Fig 1: 1) ground beef (any foodstuff containing ground meat, excepting commercial hamburgers), 2) commercial hamburgers, and 3) intact beef cuts.

The model was implemented in Microsoft Excel 2016 with the @Risk add-on package (version 7.5, Palisade Corporation, New York, USA) using inputs derived from data collected in Argentina and information gathered from experts, whenever possible. A Monte Carlo simulation with Latin Hypercube Sampling was used to assess all potential scenarios. Each simulation performed 5,000 iterations of the model, which allowed to achieve an adequate level of convergence (<1%). Model outputs were estimated as risk per serving of contaminated beef and population risk (median and 95.0% confidence intervals). To analyze the validity of the model, the predicted number of HUS cases was compared with data reported in the Argentinean Epidemiological Surveillance System [44].

### 2.2. Hazard identification

For the purpose of this study, all STEC were included in the model, assuming a similar pathogenic potential. Data of STEC prevalence at different production stages of the beef supply chain in Argentina were obtained by screening results of *stx* genes and/or STEC isolation reported in the literature (S2–S5 Tables).

### 2.3. Hazard characterization

A dose-response model was used to describe the relationship between the ingested dose of STEC from beef consumption and the probability of health endpoints of interest. The probability of illness (*P_ill_*) was estimated using a Beta-Poisson model relating the ingested dose of the pathogen and the probability of illness [45,46]. The variability in *α* and *β* parameters was modelled using PERT distributions based on the 5%, 50% and 95% percentiles estimated by Teunis et al. [45].

The probability of evolution to HUS (*P_HUSǀill_*) of all STEC cases (3.0–9.0%) and HUS mortality rate (*P_mortǀHUS_*) (2.6% in children and 12.0% in adults) were estimated from the data reported by Exeni in Argentina [46].

### 2.4. Exposure assessment

The five production modules of the beef supply chain were characterized by inputs (Fig 1). They were connected so that output distributions from each module served as inputs to the next module or as final outputs of the estimated ingested STEC dose (CFU) per serving portion (Table 1).

**Table 1:** Input parameters used in the risk assessment model of STEC due to beef consumption.

****Variable****	****Symbol****	****Unit****	****Equation/Distribution****	Reference
***1*. *Cattle primary production***				
Proportion of animals slaughtered in different seasons (autumn-winter *vs*. spring-summer)	*P_(Se)_*	Probability	~*Beta*[(6751434+1);(13468819−6751434+1)]	[47]
Proportion of animals slaughtered according to age (<18 months *vs*. >18 months)	*P_(Age)_*	Probability	~*Beta*[(8037782+1);(13468819−8037782+1)]	[48]
Proportion of animals slaughtered according to the production system (feedlot *vs*. semi-intensive system)	*P_(PS)_*	Probability	~*Beta*[(3651421+1);(13468819−3651421+1)]	[48]
*stx* prevalence in animals slaughtered in autumn-winter, <18 months and from feedlot production systems	*P_(1)_*	Probability	~*Beta*(38+1;95−38+1)	[38,49–51]
*stx* prevalence in animals slaughtered in autumn-winter, <18 months and from semi-intensive production systems	*P_(2)_*	Probability	~*Beta*(36+1;166−36+1)	[38,51,52]
*stx* prevalence in animals slaughtered in autumn-winter, >18 months and from feedlot production systems	*P_(3)_*	Probability	~*Beta*(0+1;6−0+1)	[38,51]
*stx* prevalence in animals slaughtered in autumn-winter, >18 months and from semi-intensive production systems	*P_(4)_*	Probability	~*Beta*(592+1;1980−592+1)	[38–40,47,49,51,53]
*stx* prevalence in animals slaughtered in spring-summer, <18 months and from feedlot production systems	*P_(5)_*	Probability	~*Beta*(7+1;61−7+1)	[38,49,51]
*stx* prevalence in animals slaughtered in spring-summer, <18 months and from semi-intensive production systems	*P_(6)_*	Probability	~*Beta*(145+1;238−145+1)	[38,51,52]
*stx* prevalence in animals slaughtered in spring-summer, >18 months and from feedlot production systems	*P_(7)_*	Probability	~*Beta*(3+1;18−3+1)	[4,38,51]
*stx* prevalence in animals slaughtered in spring-summer, >18 months and from semi-intensive production systems	*P_(8)_*	Probability	~*Beta*(401+1;1865−401+1)	[38–40,47,49,51]
***2*. *Cattle transport***				
Change in *stx* prevalence due to transport	*Ef_(Tr)_*	*Odds Ratio*	~*PERT*(0,561;1,028;1,882)	[54–61]
*stx* prevalence in beef cattle after transport from farm to abattoir	*P_(Tr)_*	Prevalence	$\frac{\left(\mathrm{Prevalence}\times Ef(Tr)\right)}{\left((1-\mathrm{Prevalence})+(\mathrm{Prevalence}\;x\;Ef(Tr))\right)}$ Where “Prevalence” is *P_(1)_*, *P_(2)_*, …, or *P_(8)_*	
***3*. *Processing and storage in the abattoir***				
Type of abattoir (applying HACCP-STEC vs. not applying HACCP-STEC)	*Abatt*		~*Bernoulli*(0,38)	[62]
Change in *stx* prevalence due to slaughter in abattoirs not applying HACCP-STEC	*TT_(A-noH)_*	*Odds ratio*	$\frac{∼BETA(217+1;401-217+1)}{PTr}$	[24,43,63,64]
*stx* prevalence in beef carcasses slaughtered in abattoirs not applying HACCP-STEC	*P_(c-noH)_*	Prevalence	$\frac{\left(P(Tr)\times TT(A-noH)\right)}{\left((1-P(Tr))+(P(Tr)\times TT(A-noH))\right)}$	
Change in *stx* prevalence due to slaughter in abattoirs applying HACCP-STEC	*TT_(A-H)_*	*Odds ratio*	$\frac{∼BETA(625+1;3027-625+1)}{PTr}$	[38,41,42,65]; Brusa et al. (unpublished work)
*stx* prevalence in carcasses slaughtered in abattoirs applying HACCP-STEC	*P_(c-H)_*	Prevalence	$\frac{\left(P(Tr)\times TT(A-H)\right)}{\left((1-P(Tr))+(P(Tr)\;x\;TT(A-H))\right)}$	
STEC concentration in carcasses slaughtered in abattoirs not applying HACCP-STEC	*C_(A-noH)_*	Log cfu/100cm^2^	~*Normal*(3,1;0,71(*Truncated*(1,4;5,0)))	[43]
STEC concentration in carcasses slaughtered in abattoirs applying HACCP-STEC	*C_(A-H)_*	Log cfu/100cm^2^	~*Normal*(2,367;0,89(*Truncated*(0,18;5,06)))	[42]
Storage temperature in abattoirs not applying HACCP-STEC	*Temp_(A-noH)_*	°C	~*PERT*(1;4;11)	[43]
Storage temperature in abattoirs applying HACCP-STEC	*Temp_(A-H)_*	°C	~*PERT*(0;1;3)	Industry communication
Storage time in abattoirs not applying HACCP-STEC	*Ti_(A-noH)_*	h	~*Triangular*(24;52;192)	[43]
Storage time in abattoirs applying HACCP-STEC	*Ti_(f-H)_*	h	~*Triangular*(24;27;30)	Industry communication
STEC growth during the storage period	*C_(stg)_*	Log cfu/100cm^2^	$c(stg)=C(A)+\alpha (t)-\ln\left[1-\frac{1-e^{\alpha (t)}}{e^{Ym-C(A)}}\right]$	[66]
			where: $\alpha (t)=\mu \times Temp(A)+\frac{\mu }{k}\times \left[e^{-kTemp(f)}-1\right]$	
			$k=0,00658+\frac{1.941}{1+\exp[-0.8137\times (T\mathrm{emp}(A)-22.4)]}$	
			*Ym* = 8.53×[1−*exp*(−0.108×*Temp*(*A*))]	
			$\sqrt{\mu }=∼\mathrm{Normal}(0.0901;0.004)\times (T(fA)-(∼\mathrm{Normal}(6;1)))$	
Change in *stx* prevalence due to deboning process	*OR_(deb)_*	*Odds ratio*	$\frac{BETA(178+1;2683-178+1)}{P(c-H)\;or\;P(c-no-H)}$	[41]
*stx* prevalence in beef cuts	*P_(bcA)_*	Prevalence	$\frac{\left(P(cH)\times OR(deb)\right)}{\left((1-P(cH))+(P(cH)\times OR(deb))\right)}$	
Storage temperature	*Temp_(bc)_*	°C	~*PERT*(0.2;0.4;0.5)	Industry communication
Storage time	*Ti_(bc)_*	Hours	~*Uniform*(7;15)×24	Industry communication
STEC growth in beef cuts during storage	*C_(bc)_*	Log cfu/cm^2^	Growth equation reported by Huang et al.	[66]
Surface area per gram of beef cuts	*Sa*	cm^2^/g	~*Uniform*(0,1;0,5)	[67]
Grams in 100 cm^2^ of beef cuts	*Gcm^2^*	Grams	$\frac{100}{Sa}$	
STEC concentration in beef cuts in the abattoir	*C_(bcA)_*	cfu/g	$\frac{C(bcA)}{Gcm2}$	
***3*.*b*.*- Commercial hamburger***				
Change in *stx* prevalence due to trimming process	*OR_(trm)_*	*Odds ratio*	$\frac{∼BETA(45+1;638-45+1)}{∼BETA(42+1;806-42+1)}$	[41]
*stx* prevalence in trimmings	*P_(trm)_*	Prevalence	$\frac{\left(P(cH)\times OR(trm)\right)}{\left((1-P(cH))+(P(cH)\times OR(trm))\right)}$	
Storage temperature	*Temp_(h)_*	°C	~*PERT*(−25;−20;−10)	Industry communication
Storage time	*Ti_(h)_*	Hours	~*Uniform*(2;5)×24	Industry communication
STEC growth during storage	*C_(h)_*	Log cfu/cm^2^	Growth equation reported by Huang et al.	[66]
STEC concentration at abattoir	*C_(hA)_*	cfu/g	$\frac{C(h)}{Gcm2}$	
***4*.*Retail***				
***4*.*a*.*- Beef cuts***				
Probability of washing hands (butchers)	*P_(wh)_*	Probability	~*Beta*(1+1;86−1+1)	[22]
Probability of washing the cutting board and table	*P_(wcb)_*	Probability	~*Beta*(19+1;86−19+1)	
Concentration change due to hand washing	*R_(wh)_*	%	10^~*Normal*(−0.2;1.42;*Truncated*(2))^	
Transfer rate of STEC from beef cuts to butcher´s hands	*T_(bcH)_*	%	10^~*PERT*(−0.44;0.59;2)^	[68]
STEC concentration change in unwashed hands	*p_(nonWH)_*	cfu	(*C*(*bc*)×*T*(*bcH*))/100	
STEC concentration change in washed hands	*p_(WH)_*	cfu	(*p*(*nonWH*)×*R*(*wh*))/100	
Transfer rate of STEC from hands to faucet	*T_(HF)_*	%	10^~*PERT*(−2.59;−1.08;1.09)^	
Number of STEC in faucet	*p_(F)_*	cfu	(*p*(*nonWH*)×*T*(*HF*))/100	
Transfer rate of STEC from faucet to hands	*T_(FH)_*	%	10^~*PERT*(−1.7;0.169;2)^	
Number of STEC in washed hands	*p_(WH)_*	cfu	[(*p*(*F*)×*T*(*FH*))/100]+*p*(*WH*)	
Transfer rate of STEC from hands to beef cuts	*T_(Hbc)_*	%	10^~*PERT*(−2.54;0.21;2)^	
Number of STEC in beef cuts	*p_(bc)_*	cfu	In washed hands: ((*p*(*WH*)×*T*(*Hbc*))/100)	
			In unwashed hands: ((*p*(*nonWH*)×*T*(*Hbc*))/100	
Transfer rate of STEC from beef cuts to cutting board and table	*T_(bcCB)_*	%	10^~*PERT*(0.48;1.05;1.49)^	[68]
Number of STEC in unwashed cutting board and table	*p_(CB)_*	cfu	(*C*(*CB*)×*T*(*bcCB*))/100	
Transfer rate of STEC from cutting board and table to beef cuts	*T_(CBbc)_*	%	10^~*PERT*(−0.79;−0.43;1.73)^	
Number of STEC in unwashed cutting board and table	*p_(bcnonW)_*	cfu	(*p*(*CB*)×*T*(*bcnonW*))/100	
Final number of STEC in beef cuts at butcher shops	*C_(bcB)_*	cfu	*C*(*bc*)+*p*(*bc*)+*p*(*bnonW*)	
Storage temperature at butcher shops	*Temp_(B)_*	°C	~*Trianagular*(0;4.8;14.5)	C Adriani pers. comm
Storage time at butcher shops	*Ti_(B)_*	Hours	~*Uniform*(2;5)×24	C Adriani pers. comm
STEC concentration in beef cuts after storage	*C_(stg)_*	cfu/100cm^2^	Growth equation reported by Huang et al. [66]	
***4*.*b*.*- Ground beef***				
Change in the *stx* prevalence due to beef grinding	*OR_(bc-gb)_*	*Odds ratio*	$\frac{∼BETA(176+1;636-173+1)}{∼BETA(8+1;66-8+1)}$	[22,63,69–73]; Lopez et al. (unpublished work)
Number of STEC in ground beef	*P_(gb)_*	Prevalence	$\frac{\left(P(bcA)\times TT(bc-gm)\right)}{\left((1-P(bcA))+(P(bcA)\;x\;TT(bc-gm))\right)}$	
Probability of washing mincing machine	*P_(Wmm)_*	Probability	~*BETA*(0+1;86−0+1)	
Transfer rate of STEC from beef cuts to mincing machine	*T_(bc-mm)_*	%	10^(~*PERT*(0.48;1.05;1.49))^	
Number of STEC in unwashed mincing machine	*p_(nonwmm)_*	cfu	$\frac{\left(C(stg)\times T(bc-mm)\right)}{100}$	
Transfer rate of STEC from mincing machine to ground beef	*T_(mm-gb)_*	%	10^(~*PERT*(−0.79;−0.49;1.72))^	
Number of STEC in ground beef	*p_(gb)_*	cfu	In washed mincing machine: 0	[22,68]
			In unwashed mincing machine: $\frac{\left(p(nonwmm)\times (mm-gm)\right)}{100}$	
Final number of STEC in ground beef (cm)	*C_(cm)_*	cfu	*c*(*stg*)+*p*(*gm*)	
STEC concentration in ground beef	*C_(gb)_*	cfu/g	C(cm)100∼Uniform(0.1;0.5)	
***4*.*c*.*- Commercial hamburger***				
Type of retail where hamburgers are sold	*Ret_(Hamb)_*		~*Discret*{(*supermarket;minimarket*,*butcher*); (1636;27;1069)}	S6 Table. Survey of Argentinean beef consumption habits
Type of storage in each retail	*Stg_(Ret)_*		Supermarket: ~*Discret*{(*refrigerated*;*freezing*);(195;1001)}	
			Minimarket: ~*Discret*{(*refrigerated*;*freezing*);(26;92)}	
			Butcher: ~*Discret*{(*refrigerated*;*freezing*);(411;276)}	
Storage time	*Ti_(Ret)_*	Hours	Freezing: ~*Discret*{(0,1,2,4,6,14); (228;37;602;543:385;737)}×24	
			Refrigerated: ~*Discret*{(0,1,2,4,6,14); (195;22;231:34;23;26)}×24	
STEC concentration in commercial hamburgers at retail	*C_(HRet)_*	cfu	Growth equation reported by Huang et al.	[66]
Final STEC concentration in commercial hamburgers at retail	*C_(Hg)_*	cfu/g	C(cm)100∼Uniform(0.1;0.5)	
***5*. *Home and consumption***				
***5*.*a*.*- Beef cuts***				
Storage at home	*Stg_(Hom)_*		~*Beta*(2832+1;5466−2832+1)	S6 Table. Survey of Argentinean beef consumption habits
Temperature of household refrigerators	*Temp_(re)_*	°C	~*Trinagular*(−1.5;6.1;16.1)	[74,75]
Temperature of household freezers	*Temp_(fr)_*	°C	~*Trinagular*(−41,1;−20,1;−2)	
STEC concentration in beef cuts at home	*C_(bchome)_*	cfu/g	Growth equation reported by Huang et al.	[66]
Probability of eating salad with beef cuts	*Salad*		~*Beta*(5430+1;5494−5430+1)	S6 Table. Survey of Argentinean beef consumption habits
Probability of preparing beef cuts before salad	*P_(bc-Sa)_*		~*Beta*(1079+1;3748−1079+1)	
Probability of washing hands (consumers)	*P_(WH)_*		~*Beta*(4485+1;5493−4485+1)	
Probability of washing cutting board	*P_(Wcb)_*		~*Beta*(3418+1;4468−3418+1)	
Change in STEC concentration due to washing hands	*R_(WH)_*	%	10^~*Normal*(−0.2;142;*Truncated*(2))^	
Transfer rate of STEC from beef cuts to hands	*T_(bc-H)_*	%	10^~*PERT*(−0.44;0.59;2)^	[68,76]
STEC concentration in unwashed hands	*p_(nonWH)_*	cfu	(*C*(*bcHome*)×*T*(*nonWH*))/100	
Number of STEC in washed hands	*p_(WH)_*	cfu	(*p*(*nonWH*)×*R*(*WH*))/100	
Transfer rate of STEC from hands to faucet	*T_(HF)_*	%	10^~*PERT*(−2.59;−1.08;1.09)^	[68,76]
Number of STEC in the faucet	*p_(F)_*	cfu	(*p*(*nonWH*)×*T*(*HF*))/100	
Transfer rate of STEC from faucet to hands	*T_(FH)_*	%	10^~*BERT*(−1.7;0.169;2)^	[68,76]
Number of STEC in washed hands	*p_(WH)_*	cfu	[(*p*(*F*)×*T*(*FH*))/100]+*p*(*WH*)	
Transfer rate of STEC from hands to salad	*T_(HSal)_*	%	10^~*PERT*(−2.54;0.21,2)^	[68,76]
Number of STEC in salad	*p_(Sal)_*	cfu	In washed hands: ((*p*(*WH*)×*T*(*HSal*))/100)	
			In unwashed hands: ((*p*(*nonWH*)×*T*(*HSal*))/100	
Transfer rate of STEC from beef cuts to cutting board	*T_(bc-cb)_*	%	10^~*PERT*(0.48;1.05;1,49)^	[68,76]
Number of STEC in unwashed cutting board	*p_(nonWcb)_*	cfu	(*C*(*bcHome*)×*T*(*bccb*))/100	
Transfer rate of STEC from cutting board to salad	*T_(cbSal)_*	%	10^~*PERT*(−0.79;−0.43;1.73)^	[68,76]
Number of STEC in salad	*p_(SanonWl)_*	cfu	(*p*(*nonWcb*)×*T*(*cbSal*))/100	
Final number of STEC in salad	*FC_(Sal)_*	cfu	*C*(*Sal*)+*p*(*Sal*)+*p*(*SalnonW*)	
Cooking preference	*P_(cooking)_*		~*Discret*({1,2,3,4,5};{0.003; 0.068; 0.179;0.174; 0.576})	S6 Table. Survey of Argentinean beef consumption habits
Cooking temperature	*Temp_(cook)_*	°C	~*Uniform*(75;90)	[77]
Cooking time	*Ti_(cook)_*	Minutes	According to the cooking preference and the beef cut thickness: Red: ~*Triangular*(6;7;15)	[78]
			Medium-Red: ~*Triangular*(8;12;16)	
			Medium-Well: ~*Triangular*(10;12;17)	
			Medium-Well done: ~*Triangular*(14;16;25)	
			Well done: ~*Triangular*(15;20;30)	
Decimal reduction	*D_(BC)_*		10^(11.22+0.18×*Temp*(*cook*))^	
Number of decimal reductions	*ND_(BC)_*		$\frac{Ti(\mathrm{cook})}{D}$	[79]
STEC concentration in ready-to-eat beef cuts	*C_(bccons)_*	cfu/g	10^(*c*(*bchome*)−*ND*)^	
***5*.*b*.*- Ground beef***				
Probability of eating salad with ground beef	*Salad_GB_*	Probability	~*Beta*(3651+1;4149−3651+1)	S6 Table. Survey of Argentinean beef consumption habits
Probability of preparing ground beef before salad	*Gb-Sal*	Probability	~*Beta*(1079+1;3748−1079+1)	S6 Table. Survey of Argentinean beef consumption habits
Probability of washing hands (consumers)	*P_(WH)_*	Probability	~*Beta*(4485+1;5493−4485+1)	S6 Table. Survey of Argentinean beef consumption habits
Probability of washing cutting board	*P_(Wcb)_*	Probability	~*Beta*(5286+1;5549−5286+1)	S6 Table. Survey of Argentinean beef consumption habits
Change in STEC concentration due to washing hands	*R_(WH)_*	%	10^~*Normal*(−0.2;1.42;*Truncated*(2))^	
Transfer rate of STEC from ground beef to hands	*T_(gbH)_*	%	10^~*PERT*(−0.44;0.59;2)^	[68,76]
STEC concentration in unwashed hands	*p_(nonWH)_*	cfu	(*C*(*Stg*)×*T*(*gbH*))/100	
Number of STEC in washed hands	*p_(WH)_*	cfu	(*p*(*nonWH*)×*R*(*WH*))/100	
Transfer rate of STEC from hands to faucet	*T_(HF)_*	%	10^~*PERT*(−2.59;1.08;1.09)^	[68,76]
Number of STEC in the faucet	*p_(F)_*	cfu	(*p*(*nonWH*)×*T*(*HF*))/100	
Transfer rate of STEC from faucet to hands	*T_(FH)_*	%	10^~*PERT*(−1.7;0.169;2)^	[68,76]
Number of STEC in washed hands	*p_(WH)_*	cfu	[(*p*(*F*)×*T*(*FH*))/100]+*p*(*WH*)	
Transfer rate of STEC from hands to salad	*T_(HSal)_*	%	10^~*PERT*(−2.54;0.21;2)^	[68,76]
Number of STEC in salad	*p_(En)_*	cfu	In washed hands: ((*p*(*WH*)×*T*(*Hsal*))/10	
			In unwashed hands: ((*p*(*nonWH*)×*T*(*HSal*))/100	
Transfer rate of STEC from ground beef to cutting board	*T_(gb-cb)_*	%	10^~*PERT*(0.48;1.05;1.49)^	[68,76]
Number of STEC in unwashed cutting board	*p_(nonWcb)_*	cfu	(*C*(*gb*)×*T*(*gbcbCmT*))/100	
Transfer rate of STEC from cutting board to salad	*T_(cbSal)_*	%	10^~*PERT*(−0.79;−0.42;1.72)^	[68,76]
Number of STEC in salad	*p_(SalnonWcb)_*	cfu	(*p*(*cb*)×*T*(*TcbSal*))/100	
Final number of STEC in salad	*FC_(Sal)_*	cfu	*C*(*Sal*)+*p*(*Sal*)+*p*(*SalnonWcb*)	
Cooking preference	*P_(cookgb)_*		~*Discret*({1,2,3,4,5);0.003; 0.011; 0.109;0.086; 0.791})	S6 Table. Survey of Argentinean beef consumption habits
Cooking temperature	*Temp_(cookgb)_*	°C	Red: 54.4°C	[80]
			Medium-Red: 58.6°C	
			Medium: 62.7°C	
			Medium-Well done: 65.6°C	
			Well done: 68.3°C	
Number of decimal reductions	*ND_(gb)_*		10.165+(0.211×*Temp*(*cookgm*)	[81]
STEC concentration in ready-to-eat ground beef	*C_(gbcons)_*		10^(*c*(*cgm*)−*D*(*gm*))^	
***5*.*c*.*- Commercial hamburger***				
Probability of eating salad with hamburger	*Salad_H_*	Probability	~*Beta*(3539+1;3858−3539+1)	S6 Table. Survey of Argentinean beef consumption habits
Probability of preparing hamburger before salad	*H-Sal*	Probability	~*Beta*(1079+1;3748−1079+1)	
Probability of washing hands (consumers)	*P_(WH)_*	Probability	~*Beta*(4485+1;5493−4485+1)	
Probability of washing cutting board	*P_(Wcb)_*	Probability	~*Beta*(5286+1;5549−5286+1)	
Change in STEC concentration due to washing hands	*R_(WH)_*	%	10^~*Normal*(0.2;1.42;*Truncated*(2))^	
Transfer rate of STEC from hamburger to hands	*T_(HH)_*	%	10^~*PERT*(0.44;0.59;2)^	[68,76]
STEC concentration in unwashed hands	*p_(nonWH)_*	cfu	(*C*(*Hg*)×*T*(*HH*)/100	
Number of STEC in washed hands	*p_(WH)_*	cfu	(*p*(*nonWH*)×*R*(*WH*))/100	
Transfer rate of STEC from hands to faucet	*T_(HF)_*	%	10^~*PERT*(−2.59;−1.08;1.09)^	[68,76]
Number of STEC in the faucet	*p_(F)_*	cfu	(*p*(*nonWH*)×*T*(*HF*))/100	
Transfer rate of STEC from faucet to hands	*T_(FH)_*	%	10^~*PERT*(−1.7,0.169;2)^	[68,76]
Number of STEC in washed hands	*p_(WH)_*	cfu	[(*p*(*F*)×*T*(*FH*))/100]+*p*(*WH*)	
Transfer rate of STEC from hands to salad	*T_(HSal)_*	%	10^~*PERT*(−2.54,0.21;2)^	[68,76]
Number of STEC in salad	*p_(Sal)_*	cfu	In washed hands: ((*p*(*WH*)×*T*(*HSal*))/100	
			In unwashed hands: ((*p*(*nonWH*)×*T*(*HSal*))/100)	
Transfer rate of STEC from hamburger to cutting board	*T_(Hcb)_*	%	10^~*PERT*(0.48;1.05;1.49)^	[68,76]
Number of STEC in unwashed cutting board	*p_(nonWcb)_*	cfu	(*C*(*Hg*)×*T*(*Hcb*))/100	
Transfer rate of STEC from cutting board to salad	*T_(cbSal)_*	%	10^~*PERT*(−0.79;−0.43;1.73)^	[68,76]
Number of STEC in salad	*p_(SalnonWcb)_*	cfu	(*p*(*nonWcb*)×*T*(*cbSal*))/100	
Final number of STEC in salad	*FC_(Sal)_*	cfu	*C*(*Sal*)+*p*(*Sal*)+*p*(*SalnonWcb*)	
Cooking preference	*P_(cookH)_*		*DISCRET*{(1,2,3);(0.011;0.183;0.806	S6 Table. Survey of Argentinean beef consumption habits
Cooking temperature	*Temp_(cookH)_*	°C	Medium-Red: ~*UNIFORM*(54.4;58.6)	[80]
			Medium-Well done: ~*UNIFORM*(62.7;65.6)	
			Well done: 68.3	
Number of decimal reductions	*ND_(H)_*		10.165+(0.211×*Temp*(*CookH*))	[81]
STEC concentration in ready-to-eat hamburgers	*C_(Hcons)_*		10^(*C*(*Hg*)−*D*(*H*))^	
***6*.*Consumption***				
***6*.*a*.*- Beef cuts***				
Portion size	*PS_(bc)_*	Grams	Children < 23 months: ~*LogNormal*(65.9;45.8)	Table 2. Food consumption by the Argentine population (6 months to 15 years of age) [82]
			Children 2–5 years: ~*LogNormal*(83.54;50.26)	
			Children 6–15 years: ~*LogNormal*(120.8;68.7)	
Ingested dose of STEC from beef cut consumption	*Dose_(bc)_*	cfu	With salad: (*C*(*bccons*)×*PS*(*bc*))+*C*(*Sal*)	
			Without salad: (*C*(*bccons*)×*PS*(*bc*))	
***6*.*b*.*- Ground beef***				
Portion size	*PS_(gb)_*	Grams	Children < 23 months: ~*LogNormal*(43.8;30.9)	Table 2. Food consumption by the Argentine population (6 months to 15 years of age) [82]
			Children 2–5 years: ~*LogNormal*(69.52;52.08)	
			Children 6–15 years: ~*LogNormal*(91.9;69.3)	
Ingested dose of STEC from ground beef consumption	*Dose_(gb)_*	cfu	With salad: (*C*(*gbcons*)×*PS*(*gb*))+*C*(*Sal*)	
			Without salad: (*C*(*gbcons*)×*PS*(*gb*))	
***6*.*c*.*- Commercial hamburger***				
Portion size	*PS_(H)_*	Grams	Children < 23 months: ~*LogNormal*(58.4;32.1)	Table 2. Food consumption by the Argentine population (6 months to 15 years of age) [82]
			Children 2–5 years: ~*LogNormal*(83.54;50.26)	
			Children 6–15 years: ~*LogNormal*(135.9;72.2)	
Ingested dose of STEC from hamburger consumption	*Dose_(H)_*	cfu	With salad: (*C*(*Hcons*)×*PS*(*H*))+*C*(*Sal*)	
			Without salad: (*C*(*Hcons*)×*PS*(*H*))	
***7*. *Dose-response module***				
Probability of illness	*P_(ill)_*		1−{1+(Dose)β)−α}	[45,46]
			where: *α*~*PERT*(0.000262;0.373;398.9)	
			*β*~*PERT*(0.056;39.71;39600)	
Probability of HUS	*P_(HUS)_*		~*UNIFORM*(0.03;0.09)	
Probability of death	*P_(dth)_*		~*Beta*(35+1;1302−35+1)	
Probability of HUS ǀ illness	*P_(HUSǀill)_*		*P*(*ill*)×*P*(*HUS*)	[46]
Probability of death ǀ HUS	*P_(dthǀHUS)_*		*P*(*HUS*|*ill*)×*P*(*dth*)	
***7*.*a*.*- Beef cuts***				
Number of portions	*N_(porbc)_*	Number	Children < 23 months: {(2.029.712×0.5176)×365}	Table 2. Food consumption by the Argentine population (6 months to 15 years of age) [82]
			Children 2–5 years: {(1.984.070×0.6451)×365}	
			Children 6–15 years: {(6.927.170×0.60058)×365}	
Number of cases of HUS per year due to beef cut consumption	*N_(HUSbc)_*	Number	*N*(*porbc*)×*P*(*HUS*|*ill*)	
***7*.*b*.*- Ground beef***				
Number of portions	*N_(porgb)_*	Number	Children < 23 months: {(2.029.712×0.1097)×365}	Table 2. Food consumption by the Argentine population (6 months to 15 years of age) [82]
			Children 2–5 years: {(1.984.070×0.1516)×365}	
			Children 6–15 years: {(6.927.170×0.12788)×365}	
Number of cases of HUS per year due to ground beef consumption	*N_(HUSgb)_*	Number	*N*(*porgm*)×*P*(*SHUS*|*ill*)	
***7*.*c*.*- Commercial hamburger***				
Number of portions	*N_(porH)_*	Number	Children < 23 months: {(2.029.712×0.015)×365}	Table 2. Food consumption by the Argentine population (6 months to 15 years of age) [82]
			Children 2–5 years: {(1.984.070×0.0264)×365}	
			Children 6–15 years: {(6.927.170×0.03681)×365}	
Number of cases of HUS per year due to hamburger consumption	*N_(HUSH)_*	Number	*N*(*porH*)×*P*(*HUS*|*ill*)	

#### 2.4.1. Cattle primary production

The prevalence of STEC in cattle was estimated according to three categories: a) season (spring-summer; fall-winter) [47,83], b) age of the animals (young, <18 months; adult, >18 months) [48], and c) production system (semi-intensive, feedlot) [48]. This classification resulted in eight different production scenarios (Table 1).

The proportion of animals in each age group (*P_Age_*) and season (*P_Se_*) was modelled using cattle census data corresponding to 2018 [51] (S2 Table). The probability that a slaughtered animal belonged to a feedlot or semi-intensive production system (*P_PS_*) was modelled using slaughter data from feedlot animals (S2 Table). Slaughter data from 2018 showed that the majority of animals were young (59.6%), slaughtered in spring-summer (50.1%) and from feedlots (70.4%). The probability of occurrence of the three variables (*P_Age_*, *P_Se_* and *P_PS_*) was modelled using Beta distributions.

Data describing *stx* prevalence in cattle feces were available from several peer-reviewed studies performed in Argentina (S3 Table). The combination of the three variables (*P_Age_*, *P_Se_* and *P_PS_*) allowed to model *stx* prevalence considering potential risk factors. A syllogism was used to combine the probability of occurrence of the eight level combinations (*P_1_, P_2_, P_3_, P_4_, P_5_, P_6_, P_7_,* and *P_8_*). Applying the method of moments [84], these data were used to determine parameters *α* and *β* of Beta distributions and to estimate *stx* prevalence in each combination of factors.

#### 2.4.2. Cattle transport

**Fig 2:**
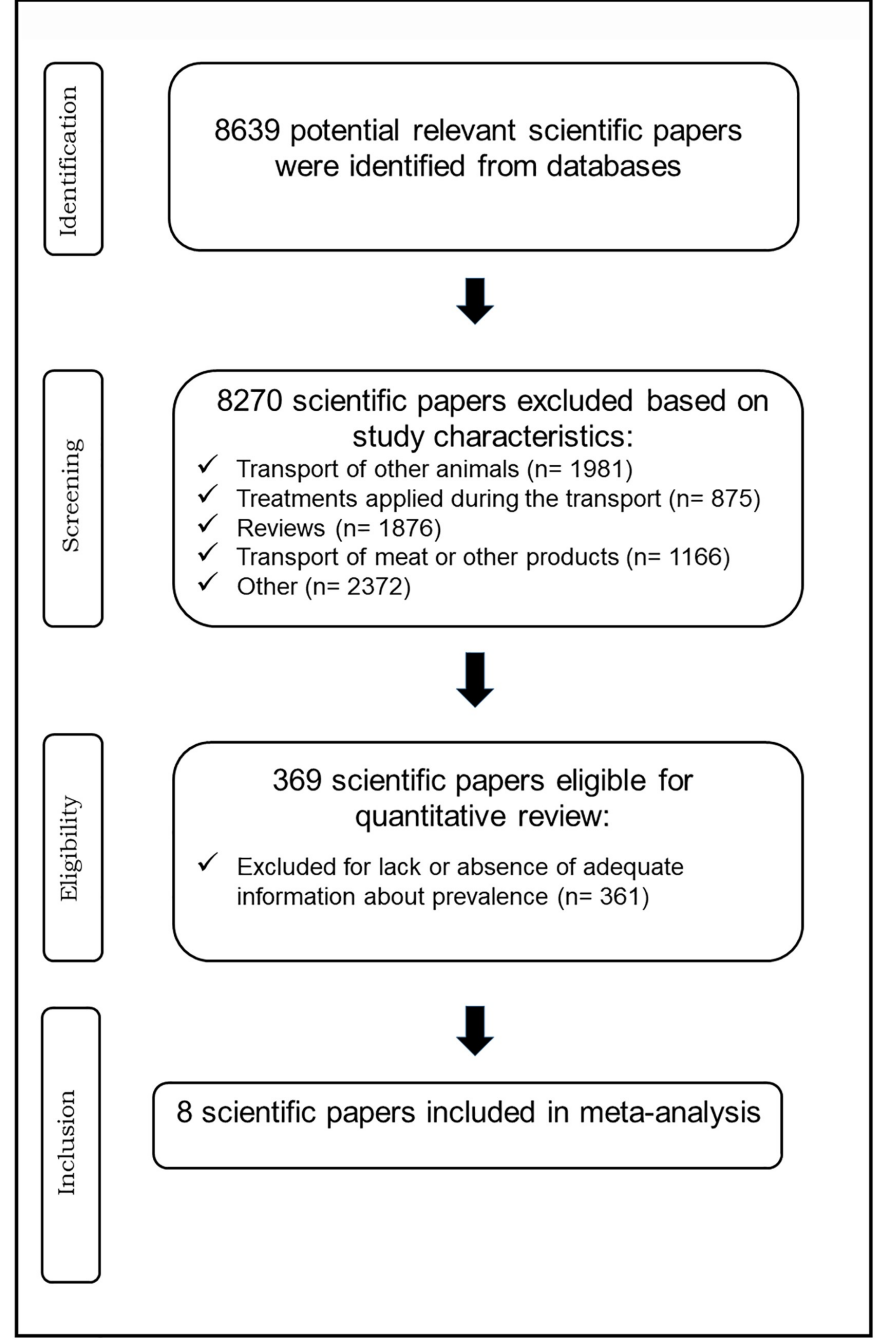
Flowchart of the cattle transport literature search according to PRISMA.

Cattle transport to abattoirs generates stress and increases cross-contamination, which could in turn modify *stx* prevalence. A systematic review and meta-analysis search of parameters related to the effect of transport on *stx* prevalence was carried out according to the Preferred Reporting Items for Systematic Reviews and Meta-Analyses (PRISMA) (Fig 2) [85]. Scopus, PubMed and Science Direct databases were searched for scientific papers unrestricted by language and published from 1980 to 2019. The research question was: “Is there evidence from the scientific literature that transport of beef cattle from farm to abattoir modifies STEC prevalence?” Search terms included “transport” AND “STEC” OR “O157:H7” OR “non-O157 STEC” OR “*stx*” AND “cattle” OR “beef cattle”. Initially, 8639 articles were identified. Abstracts and titles were assessed, selecting articles that met the *a priori* inclusion criteria. Random effect meta-analysis was performed using the Comprehensive Meta-Analysis software 2.2.064 version. Differences in *stx* prevalence in beef cattle before and after transportation were incorporated in the meta-analysis and used in the model as odds ratio (OR) values. Mean OR and 95.0% confidence interval (95.0% CI) values were used as parameters and included in a PERT distribution to model the effect of transport on STEC prevalence.

The new *stx* prevalence after transport was estimated using the transfer rate equation as follows:

$$P=\frac{Pi\times OR}{1-Pi+Pi\times OR}$$ Eq 1

where *P* is the new *stx* prevalence after a specific scenario (e.g., beef cattle in the abattoir after transport) and *P_i_* is the *stx* prevalence before the specific scenario (e.g., beef cattle in the farm) and OR is the odds ratio value between the scenarios compared. An OR less than 1 means a reduction in *stx* prevalence and an OR greater than 1 indicates an increase in *stx* prevalence [21]. This methodology was used to model the change in *stx* prevalence along the beef supply chain.

#### 2.4.3. Processing and storage in the abattoir

The prevalence of *stx* and STEC levels was modelled at various stages along the slaughtering process, from arrival of live cattle to carcass storage in the cold chamber (Fig 1, Table 1). As already mentioned, abattoirs were classified as “applying HACCP-STEC” (38.0%) and “not applying HACCP-STEC” (62.0%) [62]. The probability of slaughter in each type of abattoir was modelled using the Bernoulli distribution model (*Abatt*). Each abattoir type was modelled differently: HACCP-STEC included the production of vacuum-packaged beef cuts and commercial hamburgers all within the abattoir plant, whereas abattoirs not applying HACCP-STEC were modelled from the production of half carcasses within the plant to the transport to retail for the production of beef cuts and ground beef.

The prevalence of *stx* in carcasses varied according to the type of abattoir and was modelled using scientific publications conducted in Argentina (S4 Table). The OR value from cross-contamination during slaughtering was calculated using *stx* prevalence in carcasses and live cattle jointly for abattoirs applying HACCP-STEC (*TT_A-H_*) and not applying HACCP-STEC (*TT_A-noH_*) (S4 Table), using the previously mentioned Eq 1.

Enumeration levels of STEC were estimated by using generic *E*. *coli* counts in carcasses from abattoirs applying HACCP-STEC (*C_(A-H)_*) [42] and not applying HACCP-STEC (*C_(A-noH)_*) [43]. This was considered as the most conservative scenario as is expected STEC enumeration levels to be much lower than generic *E coli* counts. The levels of STEC during cold chamber storage (*C_(stg)_*) were estimated using the growth equation reported by Huang et al. [66]. The growth of STEC in beef cuts, commercial hamburgers and ground beef in the cold chamber and at retail was estimated using the same equation. Cold chamber temperature (*Temp_A-H_*) and storage times (*Ti_f-H_*) of HACCP-STEC abattoirs were provided by the participating plants (Industry communication). Temperature (*Temp_A-noH_*) and storage times (*Ti_A-noH_*) of abattoirs not applying HACCP-STEC were obtained from the work by Costa et al. [43] (Table 1).

**Beef cuts.** Operators, equipment, the environment and beef are sources of STEC contamination during cutting and deboning. Both operations were modelled in HACCP-STEC abattoirs only because abattoirs not applying HACCP-STEC provided half-carcasses to retails, where they were thus modelled. The OR value due to cross-contamination during deboning to obtain beef cuts (*OR_(deb)_*) was modelled with data obtained in Argentina by Brusa et al. [41] in HACCP-STEC abattoirs. The *stx* prevalence in beef cuts (*P_(bcA)_*) was calculated from the *stx* prevalence in carcasses stored in cold chambers (*P_(c-H)_*) and the OR value due to deboning (*OR_deb_*) (Table 1). The STEC concentration was estimated per 100 cm^2^ of beef cuts and considered as superficial contamination. To convert load per cm^2^ (log CFU/cm^2^) to load per gram of product (log CFU/g) (*C_bcA_*), the relationship between the two measures was estimated. According to previous estimates, a gram of beef corresponds to 0.1–0.5 cm^2^ cut surface (Sa) [37].

**Commercial hamburgers.** The transfer rate (OR) from carcasses to trimmings (*OR_(trm)_*) was estimated using data published by Brusa et al. [41] in HACCP-STEC abattoirs (Table 1). The prevalence of *stx* in trimmings (*P_(trm)_*) was estimated by combining the prevalence in carcasses stored in cold chambers and the contamination resulting from cutting and deboning. The growth of STEC in commercial hamburgers (*C_(hA)_*) was modelled using the storage temperature (*Temp_h_*) and storage time (*Ti_h_*) values provided by abattoirs (Table 1) (Industry communication).

#### 2.4.4. Retail. Beef cuts

**Fig 3:**
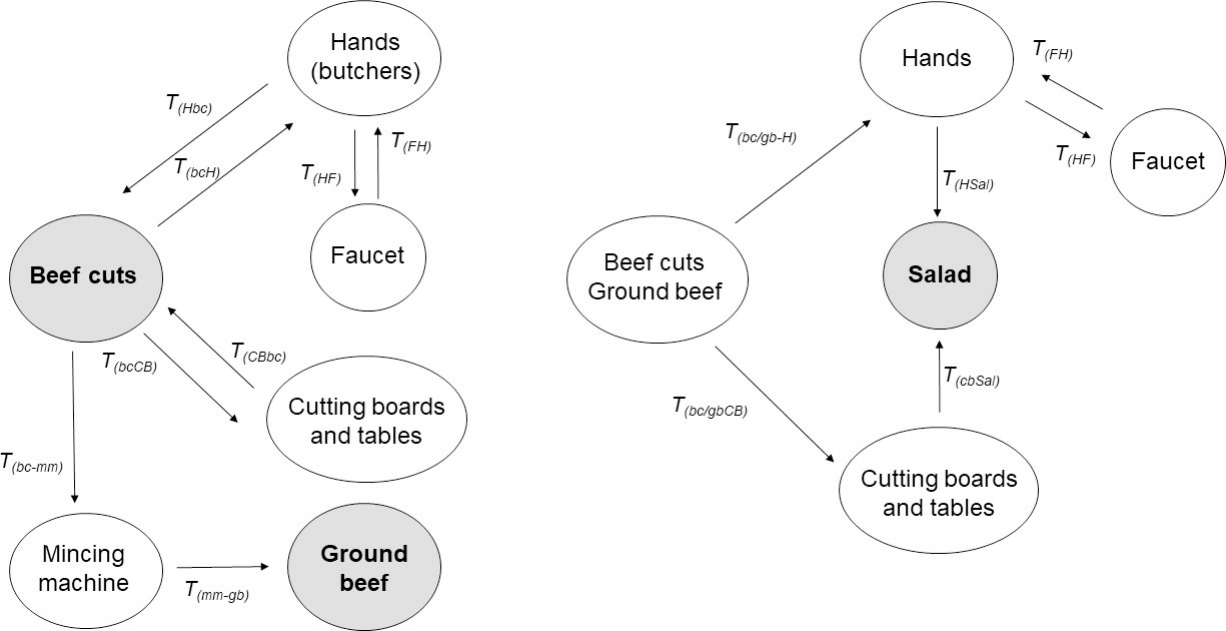
Cross-contamination scenarios at retail and home.

The cross-contamination rate during deboning to obtain beef cuts at retail was estimated using the same equation as in abattoirs applying HACCP-STEC. To incorporate cross-contamination due to retail handling, the probability of occurrence of certain practices was estimated from behavioral surveys conducted in Argentina, which included the probability of hand washing (*P_wh_*) and cutting board washing (*P_wcb_*) during beef handling [22]. Bacterial transfer rate from beef to hands (*T_(bcH)_*) and to cutting boards and tables (*T_(bcCB)_*) and reduction rate by hand washing (*R_(wh)_*) were estimated according to Montville and Schaffner [68] (Table 1, Fig 3). The growth of STEC at retail was modelled using the temperature (*Temp_(B)_*) and storage time (*Ti_(B)_*) values at retail provided by the Sanitary Authority of the city of Berisso, Buenos Aires, Argentina (C Adriani pers. comm).

**Ground beef.** The *stx* transfer rate (OR) from beef cuts to ground beef (*OR_bc-gb_*) at retail was estimated based on the *stx* prevalence in beef cuts and ground beef reported in Argentina (Table 1, S5 Table). The *stx* prevalence in ground beef (*P_gb_*) was estimated from the prevalence in beef cuts (P*_bcA_*), modified according to the estimated transfer rates resulting from handling scenarios at retail (S5 Table). The STEC concentration in ground beef (*C_gb_*) was estimated by the probability of washing the mincing machine (*P_(Wmm)_*) [22] and the bacterial transfer rate (*T_(bc-mm)_*) [68].

**Commercial hamburgers.** The proportion of commercial hamburgers (*Ret_(Hamb)_*) and conditions (*Stg_(Ret)_*) (frozen, chilled, other) in each retail outlet (mini-markets, supermarkets and butcher shops) was modelled according to consumer preferences (S6 Table). The STEC concentration in hamburgers at retail (*C_(HRet)_*) was modelled considering the storage temperature (*Temp_(h)_*) of each outlet with data from Evans and Redmond (2015) and James et al. (2017). The storage period at retail (*Ti_(Ret)_*) was modelled considering the answers provided by Argentinian consumers (S6 Table) [74,75].

#### 2.4.5. Home and consumption

Beef consumption habits in Argentina were surveyed (S6 Table) using a descriptive epidemiological design. The survey was anonymous and self-administered. It consisted of 16 closed questions with different options to evaluate frequency and preference of beef consumption, place of acquisition, habit of beef storage and preparation. Informed consent was attached regarding anonymity, non-mandatory participation and use of research results.

The growth of STEC on each beef product (beef cuts, ground beef and hamburger) during storage at home (*Stg_(Hom)_*) was modelled using the temperature values of household refrigerators (*Temp_(re)_*) and freezers (*Temp_(fr)_*) from Evans and Redmond [74] and James et al. [75].

Cross-contamination at home was modelled using the bacterial transfer rates among the different surfaces (cutting boards, hands, faucet) reported by Montville and Schaffner [68] and Chen *et al*. [68,76]. The probability that consumers prepared salads together with beef (*Salad*), hand washing (*P_(WH)_*) and cutting board washing (*P_(Wcb)_*) was estimated from the survey of Argentine consumers (S6 Table).

The effect of cooking at home on STEC concentration in beef cuts was modelled considering five cooking preferences (red, medium-red, medium-well, medium-well done, well done) (S6 Table). For each cooking preference (*P_cooking_*), cooking time (*Ti_cook_*) was estimated taking into account the time to achieve the desired beef doneness and cut thickness [78]. Cooking temperature (*Temp_cook_*) at the surface of beef cuts (where bacterial contamination is present) was estimated to vary between 75 to 90°C [77]. Log STEC reduction during cooking of beef cuts (*ND_(BC)_*) (log CFU/g) was estimated by dividing cooking time by the D-value (D*_(BC)_*) at each cooking temperature, using the D-values obtained from several *E*. *coli* O157:H7 strains isolated from beef [79]. The STEC concentration after cooking (C*_(bccons)_*) (CFU/g) was estimated by the difference between the concentration in raw beef cuts (C*_(bchome)_*) and the log reduction due to cooking (N*_D(BC)_*).

The effect of cooking during the preparation of commercial hamburgers and ground beef was modelled as a function of the final internal product temperature (*Temp_cook_*) in ground beef (P*cookgb*) and hamburgers (P*cookH*) for each cooking preference of Argentinean consumers (S6 Table). In order to compare our results with previous studies reporting the preference of consumption of ground beef and hamburgers as "pink" in the center of the mass, the categories "red" and "medium-red" of our survey were considered jointly as "pink". Each cooking preference was related to an internal temperature using the approach reported by Jackson et al. [80]. Within-variability of internal temperatures for each cooking preference was modelled using a uniform distribution. Log STEC reduction during cooking of ground beef (ND*_(gb)_*) and hamburgers (ND*_(H)_*) was estimated using the linear model reported by Juneja el al. [81]. Final STEC concentration was estimated using the same approach as explained in beef cuts.

### 2.5. Risk characterization

The QMRA model used the specific conditions for the production of each type of beef product (beef cuts, ground beef and hamburgers) under two abattoir systems in Argentina, considering the intrinsic variability and uncertainties of each process. Risk characterization was expressed as probability of illness (diarrhea due to STEC infection) and number of HUS cases after consuming STEC-contaminated beef products.

Children aged 6 months to 15 years were considered the target population of this study as they represent the age group with the highest HUS incidence in Argentina [7]. Final exposure to STEC was estimated as the combination of the ingested dose (CFU) in a beef serving (beef cuts, ground beef, hamburger) and the dose ingested during salad consumption in case both were consumed together. Portion sizes, frequency of consumption of each beef product (*Nporbc*, *Nporgb*, *NporH*) and population stratum were obtained from the National Nutrition and Health Survey of Argentina [82] (Table 2). Population estimates of each stratum were assessed in accordance with the 2010 National Census of Population, Households and Housing [86]. The number of annual HUS cases due to beef consumption (*N_(HUSbc)_*, *N*_*(HUSgb)*,_ *N_(HUSH)_*) was estimated considering the probability of acquiring the disease (*P_(HUSǀill)_*) and the frequency of beef consumption (Table 2).

**Table 2:** Beef consumption by the Argentine population (6 months to 15 years of age) [82].

Age (Population)	6–23 months (2,029,712)			2–5 years (1,984,070)			6–15 years (6,927,170)		
Foodstuff	Beef cuts	Ground beef	Commercial hamburger	Beef cuts	Ground beef	Commercial hamburger	Beef cuts	Ground beef	Commercial hamburger
**Daily consumption frequency**	0.52	0.11	0.01	0.64	0.15	0.03	0.60	0.13	0.04
**Mean portion size (g) (SD)**	65.9 (45.8)	43.8 (30.9)	58.4 (32.1)	83.5 (50.3)	69.5 (52.1)	83.5 (50.3)	120.8 (68.7)	91.9 (69.3)	135.9 (72.2)
**Total portions consumed**	383,461,310	81,270,683	11,112,673	467,172,098	109,786,529	19,118,499	1,518,516,712	323,333,972	93,071,032

### 2.6. Sensitivity analysis

Sensitivity analysis was performed using @Risk (Palisade Inc.) to identify the processing steps with the greatest impact on the risk of acquiring STEC infection and thereby identify the risk management strategies that would generate the greatest impact on public health.

## 3. Results

### 3.1. Cattle primary production

The *stx* prevalence during primary production for all production scenarios (season, age of the animals and production system) was 25.1% (6.2–64.4, 95.0% CI). Results differed when *stx* prevalence was calculated for each specific scenario, as follows: 26.2% (7.3–43.1) in fall-winter and 36.2% (9.3–64.6) in spring-summer; 36.9% (10.8–64.1) in young and 22.9% (5.1–31.5) in adult cattle; and 35.0 (3.6–45.2) and 22.4% (18.8–64.1) in semi-intensive and feedlot production systems, respectively. As it can be observed, spring-summer, young cattle and semi-intensive production system showed the highest prevalence.

### 3.2. Cattle transport

The systematic literature search yielded 30 scientific studies using the terms “*transport*”, “*beef cattle*”, “*STEC prevalence*” and “*stx prevalence*”. Reviews and prevalence studies in other animals or animals not producing food and reports with limited data to estimate *stx* prevalence before and after transport were excluded (n = 18). Twelve articles were used to estimate the impact of transport on *stx* prevalence. The estimated pooled OR was 1.0 (0.6–1.9), showing a significant heterogeneity (Q-statistic: *P*< 0.0001; *I^2^*-statistic = 91.6%).

### 3.3. Processing and storage in the abattoir

The prevalence of *stx* on carcass surfaces in abattoirs applying and not applying HACCP-STEC was 23.3 (18.8–41.6) and 42.7% (36.2–63.8), respectively. The enumeration of STEC levels was 1.7 (0.3–3.4) and 2.7 (1.3–4.2) log CFU/100 cm^2^, respectively.

### 3.4. Retail

#### 3.4.1. Beef cuts

The prevalence of *stx* and STEC concentration in beef cuts was estimated considering whether the carcass supplier applied HACCP-STEC or not. Thus, *stx* prevalence was 28.4 (19.9–49.4) and 48.8% (37.3–70.1), respectively and STEC concentration was -2.9 (-5.0 and 0.4) and -0.2 (-3.4 and 3.6) log CFU/g, respectively.

#### 3.4.2. Ground beef

Both *stx* prevalence and STEC concentration were estimated considering the available information from abattoirs applying or not HACCP-STEC and the effect of handling beef at retail. Accordingly, *stx* prevalence was 73.6% (55.8–89.3) and STEC concentration was -2.82 log CFU/g (-3.4–2.5).

#### 3.4.3. Commercial hamburgers

The model incorporated information of Argentinean abattoirs applying HACCP-STEC. Thus, *stx* prevalence in trimmings was 30.1% (20.3–52.2) and STEC concentration in hamburgers was -2.9 log CFU/g (-5.0 and 0.4).

### 3.5. Home and consumption

A total of 5,658 surveys from 23 jurisdictions in Argentina were collected in April 2019 (S6 Table). Regarding beef cuts, 89.7% of surveyed consumers acquired this product chilled at retail and 56.7% stored beef cuts frozen at home. Most consumers (99.7%) preferred levels of cooking that ensured STEC removal from the surface of beef cuts. The most preferred levels of cooking were "well-done" (57.6%), “medium-well done” (17.4%) and “medium-well” (17.9%). In the case of ground beef, 46.6% of people acquired the product chilled at retail and 49.8% stored ground beef frozen at home. The preferred level of cooking was “well-done” (79.1%) followed by “medium-well” (10.9%). Finally, commercial hamburgers were obtained frozen at retail (56.9%), stored at home once frozen (78.0%), and people preferred them “well-done” (80.6%) and “medium-well” (9.9%).

According to the type of side dish, 45.1–66.8% of surveyed individuals preferred the consumption of any beef product along with fresh vegetables; 51.8% reported having two separate tables to prepare beef and vegetables, whereas 15.8% used the same table for both, always washing the table with detergent in between handling these foods. After handling beef, 16.2% of consumers reported to wash their hands and 4.2% reported to wash the utensils.

The STEC concentration in raw beef cuts, ground beef and commercial hamburgers was 1.3 (-3.4–3.4), -2.7 (-3.4–3.9) and -2.8 (-3.4–3.0) log CFU/g, respectively. The STEC transfer rates from beef cuts, ground beef and commercial hamburgers to salad was -5.0 (-5.0–3.9), -5.0 (-5.0–0.5) and -5.0 (-5.0–0.9) log CFU/g, respectively.

### 3.6. Risk characterization

Median HUS probability from consumption of beef cuts, ground beef and commercial hamburgers was <10^−15^ (<10^−15^–6.0x10^-3^, 90.0% CI), 5.4x10^-8^ (3.5x10^-10^–3.9x10^-4^) and 3.5x10^-8^ (3.0x10^-10^–2.0x10^-4^), respectively (Table 3). The expected average annual number of HUS cases from consumption of beef cuts, ground beef and commercial hamburgers was 0, 28 and 4, respectively. The expected annual number of deaths due to ground beef and commercial hamburger consumption was 2 and 0, respectively.

**Table 3:** Probability of illness, HUS and death and annual number of HUS cases from consumption of beef cuts, ground beef and commercial hamburgers contaminated with STEC.

Foodstuff	Probability*			Expected median HUS cases per year
	Illness	HUS	Mortality	
**Beef cuts**	<10^−15^ (<10^−15^–8.0x10^-2^)	<10^−15^ (<10^−15^–6.0x10^-3^)	<10^−15^ (<10^−15^–7.9x10^-4^)	0
**Ground beef**	9.0 x 10^−7^ (6.3x10^-9^–7.0x10^-3^)	5.4x10^-8^ (3.5x10^-10^–3.9x10^-4^)	6.4 x 10^−9^ (4.2x10^-11^–4.7x10^-5^)	28
**Commercial hamburgers**	5.8x10^-7^ (8.2x10^-9^–4.1x10^-3^)	3.5 x 10^−8^ (3.0x10^-10^–2.0x10^-4^)	4.2x10^-9^ (5.4x10^-11^–2.9x10^-5^)	4

*Median (90% CI).

### 3.7. Sensitivity analysis

#### 3.7.1. Beef cuts

**Fig 4:**
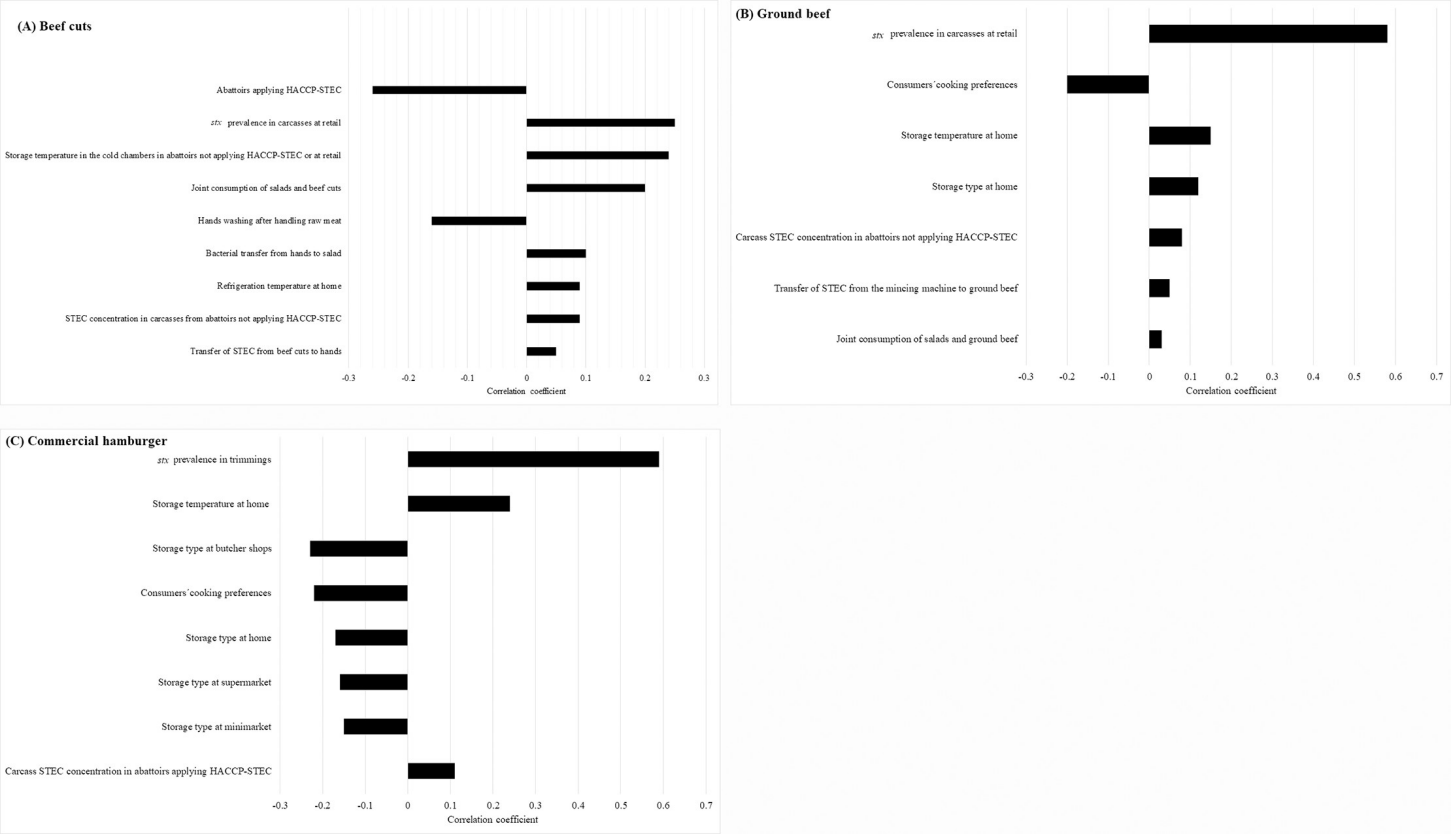
(A-C). Sensitivity analysis of model inputs on the probability of developing HUS.

The risk of STEC infection from beef cut consumption and subsequent outcomes correlated with abattoirs applying HACCP-STEC, *stx* prevalence in carcasses at retail, storage temperature in cold chambers of abattoirs not applying HACCP-STEC or at retail, joint consumption of salad and beef cuts, hand washing after handling raw meat, transfer of STEC from hands to salad, refrigeration temperature at home, STEC concentration in carcasses from abattoirs not applying HACCP-STEC, and bacterial transfer from beef cuts to hands (Fig 4(A)).

The most significant input for the risk of STEC infection was the type of abattoir for beef production. This model input negatively correlated with the risk of infection (the higher the percentage of abattoirs applying HACCP-STEC, the lower the probability of illness). Such effect may be explained by the lower *stx* prevalence and STEC concentration in carcasses produced in abattoirs applying HACCP-STEC (28.4%; mean concentration, -2.9 log CFU/g) as compared with abattoirs not applying HACCP-STEC (48.8%; mean concentration, -0.2 log CFU/g). Thus, consumers eating beef cuts produced in an abattoir not applying HACCP-STEC had 1.7 times higher probability of being exposed to STEC as compared with abattoirs applying HACCP-STEC.

Likewise, hand washing negatively correlated with the probability of infection, proving the impact of this practice on disease occurrence. Storage temperature in the abattoir, at retail and home had a great influence on the probability of infection, with a 3.5 and 7.4 times increased risk of HUS if beef cuts were stored at 8 and 10°C, respectively.

#### 3.7.2. Ground beef and commercial hamburgers

The risk of STEC infection from ground beef and commercial hamburger consumption and subsequent outcomes correlated positively with *stx* prevalence in carcasses at retail and trimmings in the abattoir, storage temperature at home, storage type at home, carcass STEC concentration in abattoirs not applying HACCP-STEC (ground beef) and applying HACCP-STEC (commercial hamburgers), transfer of STEC from the mincing machine to ground beef, and joint consumption of salad and ground beef (Fig 4(B) and 4(C)). The probability of HUS was 1.2 times higher if ground beef was elaborated with beef provided by abattoirs not applying HACCP-STEC. Ground beef cooking preference was the only input with a negative correlation, i.e., the higher the percentage of consumers who preferred a higher degree of beef doneness (well-done was selected by 79.1% of consumers), the higher the STEC reduction after cooking and the lower the probability of infection. The positive correlation between STEC transfer from the mincing machine to ground beef and the probability of acquiring HUS due to ground beef consumption evidenced the impact of good hygiene practices (GHP) at retail.

## 4. Discussion

This risk assessment study allowed to shed light into the potential role of beef consumption in the development of HUS cases in the Argentinean child population, considering the very limited epidemiological information on food sources in the country [6]. The QMRA included all the available information throughout the Argentinean beef production chain, from primary production to home consumer habits [38–43,50]. Although a risk assessment of HUS from hamburger consumption had already been carried out in Argentina [37], the relevance of the current QMRA is concerned with the inclusion of new information that responds to the uncertainties identified in the previous risk assessment [37], such as a) risk factors associated with the presence of STEC in primary production, b) effect of the transport of live animals, c) identification of abattoirs with different risk levels, d) evaluation of the effect of cross-contamination in butcher shops, e) application of a survey to assess beef consumption habits at home at national level, not just regional, and f) consideration of other meat matrices. Despite the quantity and quality of the information used in the current QMRA were better, the risk of HUS from hamburger consumption was very similar in both models (3.5x10^-8^ *vs*. 4.6x10^-8^).

### 4.1. Cattle primary production

Cattle are the major STEC reservoir, and beef has been essentially identified as the main vehicle associated with the transmission of this group of microorganisms [52]. The mean *stx* prevalence in cattle estimated by the QMRA (25.1%, 6.2–64.4%, 95.0% CI) was in the range of that reported in Brazil, USA, Italy and Spain (21.3–36.2%) [87–91]. The STEC prevalence reported in studies conducted in Argentina is also within the same range (11.8–38.9%) [38,39,52,53]. However, other authors have reported higher mean STEC prevalence in cattle feces in Paraguay, Canada, Germany, Ireland, UK, France and Australia (44.8–84.8%) [92–99]. International studies have identified differences in cattle *stx* prevalence according to season, cattle age and feeding practices [48,83,87,97,100,101]. However, the QMRA model did not show any statistical association between these primary production variables and the risk of developing HUS from any beef product. More detailed prevalence studies including different production scenarios in Argentina could validate the model conclusions.

### 4.2. Cattle transport

The impact of transport on STEC prevalence in cattle is controversial. In this study, the pooled OR impact of transport on STEC prevalence in cattle was 1.0 (0.6–1.9). Other studies have observed an increase [57,102,103], a reduction [58], no change [60,61,101] and even contradictory results [54,104] in the prevalence and spread of STEC in bovine faeces caused by transport.

### 4.3. Processing and storage in the abattoir

Carcass contamination with STEC can occur during the slaughtering process in the abattoir, and STEC-contaminated carcasses can carry over the contamination to beef cuts and trimmings [41,42,63]. The prevalence of *stx* in carcasses was 23.3% (18.8–41.6) in HACCP-STEC abattoirs and 42.7% (36.2–63.8) in abattoirs not applying HACCP-STEC. A similar or higher prevalence has been reported in the USA (23.0 and 60.6%) and UK (27.0%) [95,105,106]. In Canada, the proportion of STEC confirmed by isolation from carcasses was 5.4% [107]. In Argentina, the isolation rate of STEC strains was 5.8 to 9.0% in abattoirs applying HACCP-STEC [24,38,41].

The concentration of STEC in carcasses was also associated with the type of abattoir. The probability of developing HUS from beef cut consumption was lower (1.7x) if carcasses were provided by abattoirs applying HACCP-STEC, evidencing the impact of targeting the food safety mitigation strategies against STEC. In these abattoirs, beef cuts are vacuum-packaged, avoiding later product contamination until consumption. On the other hand, abattoirs not applying HACCP-STEC do not cut and debone carcasses; these processes are performed in butcher shops that do not even apply GMP [22]. As the type of abattoir was one of the most influential model inputs on the risk of HUS, the use of a single sanitary standard (application of HACCP-STEC) in all Argentinean abattoirs and during transportation of packaged beef cuts would have the greatest impact on HUS reduction.

### 4.4. Retail

The prevalence of *stx* in beef cuts at retail was also higher if carcasses were produced in abattoirs not applying HACCP-STEC (48.8 vs. 28.4%). Studies conducted in Uruguay and the USA have reported 28.0% and 36.0% *stx* detection in beef cuts, respectively [105,108]. In Argentina, *stx* detection in retail beef cuts was 12.1% [63], and even lower in Chile, Brazil, Canada and Italy (0.7–8.4%) [109–111].

Food products elaborated with ground beef are considered an epidemiologically important source of STEC infections due to contamination spread during mincing [22,112]. Although the *stx* prevalence estimated by the QMRA in ground beef at retail (73.6%) was similar to that reported in Chile, Brazil, USA, Italy, Spain and Australia (2.1–49.3%) [113–120], studies conducted in Argentina have reported a lower prevalence (6.1–45.3%) (S5 Table). Differences may be due to true differences in STEC shedding rates in cattle, GMP and HACCP practices in the abattoir and storage conditions at retail. It is important to note that the laboratory methodologies or criteria (screening or isolation) to consider positivity for STEC differed, which may also account for differences in prevalence levels between studies.

Commercial hamburgers are elaborated with beef trimmings obtained from deboning in abattoirs applying HACCP-STEC. The *stx* prevalence in trimmings was 30.1%, including activities that could lead to cross-contamination (slaughtering, quartering, deboning). In this regard, the only study conducted in Argentina reported 1.4% *stx* prevalence in trimmings [41], whereas studies in New Zealand, Australia, USA and Uruguay informed a higher *stx* prevalence (9.7–30.0%) [108,121].

### 4.5. Risk characterization

#### 4.5.1. Beef cuts

In the present study, the mean probability of illness, HUS and death from beef cut consumption in children under 15 years was <10^−15^, with an expected number of zero HUS cases per year (95.0% CI 0–0). In a risk assessment carried out in Canada [30], the mean probability of illness (2.9x10^-9^) from beef cut consumption was six orders of magnitude greater than in our study. In our QMRA, storage temperature at retail (>5°C) was a risk variable for HUS development due to beef cut consumption, as identified in the sensitivity analysis. Application of GMP along the beef chain and storage of beef at temperatures below 5°C were identified as protective factors against HUS. Since microbial contamination in beef cuts is superficial and STEC are not heat-resistant, exposure to recommended cooking temperatures eliminates STEC [122]. In Argentina, children prefer beef cuts to ground beef and commercial hamburgers (Table 2) [82]. Even though most Argentinean consumers (99.7%) prefer eating beef cuts "medium-red" to "well-done", the sensitivity analysis did not identify the level of cooking as a factor that impacted on HUS risk.

#### 4.5.2. Ground beef and commercial hamburgers

The mean probability of illness, HUS and death from ground beef consumption in Argentine children under 15 years was 9.0x10^-7^, 5.4x10^-8^ and 6.4x10^-9^, respectively, and 5.8x10^-7^, 3.5x10^-8^ and 4.2x10^-9^, respectively, from commercial hamburger consumption. The expected annual number of HUS cases from ground beef and commercial hamburger consumption was 28 and 4, respectively. The present HUS QMRA is similar to other risk assessments developed in Canada [30,33], Australia [34], the Netherlands [123], USA [35,124], Ireland [27] and Argentina [37], all of which considered primary production conditions, distribution, storage and consumption. The probability estimates reported in those studies (P*_illness_*, 6.0×10^−7^–1.8×10^−4^), (P*_HUS_*, 4.2×10^−9^–6.4×10^−5^) and (P*_death_* 5.9×10^−10^–2.3×10^−6^) were within the values informed here. In a previous risk assessment carried out in Argentina [37], the probability of HUS from home-made and commercial hamburger consumption was 4.6x10^-8^ (95.0% CI, 7.4x10^-11^–1.6x10^-4^), similar to the one obtained with the present QMRA. In agreement with a study conducted in Canada, home storage conditions were a protective factor against HUS from ground beef consumption [30]. On the other hand, cross-contamination at retail, specifically the transfer of STEC from the mincing machine to ground beef due to lack of standardized sanitation operating procedures (SSOP) and GHP, significantly increased bacterial loads and the public health risk associated with ground beef consumption [22,125]. In Argentina, most consumers (70.0%, S6 Table) purchase ground beef in butcher shops, the majority of which do not apply SSOP, GHP or GMP [22]. The probability of HUS was 1.2 times higher if ground beef was elaborated with carcasses provided by abattoirs not applying HACCP-STEC. Thus, applying HACCP-STEC in all abattoirs could help reduce HUS incidence. In this context, it would be interesting to evaluate the impact of HACCP-STEC from ground beef production to immediate packaging after processing.

The *stx* prevalence in trimmings was also associated with higher risk of HUS from commercial hamburger consumption. Storage at refrigeration temperatures (<5°C) at retail and home were protective factors against HUS. In agreement with other risk assessments, cooking was the most influential model input for ground beef and hamburgers [27,30,33–36]. Opposite to other survey studies conducted in Ireland and Norway reporting 65.0% and 45.7% of consumers eating hamburgers well-done [126,127], most consumers in Argentina preferred eating ground beef (79.1%) and commercial hamburgers (80.6%) well-done. Such preference for a higher degree of meat doneness was seen as a protective factor against the risk of acquiring HUS.

Differences in the probabilities estimated by the different models worldwide reflected the diverse conditions of food production, distribution, storage and preparation [36]. However, all models were markedly similar in terms of the factors having the highest risk impact. The prevalence and concentration of the pathogen in faeces and carcasses and the cooking temperature of beef were the most influential variables in all the published models.

The cross-contamination module “at home” regarding Argentinean habits was incorporated to capture the effect of food preparation practices on disease transmission. Storage temperature was identified in the sensitivity analysis of all beef products of our model. This coincided with other authors [123] and reinforced the idea of the impact of storage and processing practices at home on the risk of HUS. Cross-contamination has been previously proposed as a factor associated with illness and increased HUS risk [27,36,67]. Vegetables have been associated with STEC cases and outbreaks worldwide [19,128–133], and STEC cross-contamination from beef to vegetables as well as the effect of hygiene measures have also been studied [134–136]. In our QMRA, the joint consumption of salads with beef was identified as a risk factor for HUS due to improper hygiene practices at home and vegetable contamination from meat, although the effect of the possible level of STEC contamination of vegetables was not included. Other QMRA did not consider or identify the joint consumption of salads with beef as a risk factor for HUS. The sensitivity analysis of all foodstuffs in our model estimated that the impact of consumers´ habits during food preparation at home was lower than that of variables such as type of abattoir, *stx* prevalence in carcasses or storage of beef at retail. However, their influence on the probability of HUS should not be underestimated.

### 4.6. Is beef consumption the only responsible for endemic HUS in Argentina?

Haemolytic-uremic syndrome is considered a multifactorial disease [18] and, for this reason, HUS endemicity in Argentina cannot be explained only by beef consumption. Although the consumption of raw beef, raw milk, lettuce, sprouts, fruit juices and vegetables is recognized as a potential source of STEC infection in human beings [137], environmental exposure, direct contact with animals and person-to-person transmission have also been identified as important risk factors [18,138–141]. In Argentina, information on potential food sources and transmission routes other than beef is scarce. However, an epidemiological study showed that eating undercooked beef outside home, living or visiting a place with pets and being in contact with children <5 years old with diarrhoea were risk factors for HUS [142]. The routes of transmission have expanded from direct or indirect contact with cattle or animal food products to include direct contact with infected people that may be actively shedding STEC [18].

The rate of HUS cases reported in Argentina ranges from 300 to 500 new cases per year, with a median of 349 cases in the period 2010–2016 [6]. The average number of annual HUS cases in this study was 32, all related to the consumption of beef products. On average, 10.0% of HUS cases reported in children under 15 years in Argentina would be due to beef consumption, especially ground beef. Official reports of the period 2002–2015 only attributed 0.1–0.06% of cases to beef consumption [6]. The last epidemiological report in Argentina has shown a slight decrease in HUS cases, totalling 290 cases [7]. Such tendency could be explained by consumers’ habits, the improvements implemented along the beef production chain and specific legislation on beef products. However, HUS primarily affects 1-year-old children. The annual rate slightly increased from 12.3 cases per 100,000 in 2018 [44] to 12.9 cases in 2019 [7]. According to the Argentinian National Nutrition and Health Survey [82], beef cuts are the beef food most consumed by this sub-population. In the present evaluation, no HUS cases from beef cut consumption would be expected. In this context, other potential sources of infection should be included to implement actions tending to reduce HUS in the affected sub-population. For example, in 2011–2015, 39 HUS outbreaks were reported in Argentina; 30 were associated with home origin, 5 with kindergarten and 4 with the community [6]. Fernandez Brando et al. [17,143] reported that 75.0% of children in urban and suburban areas and 68.7% of healthy adults working in kindergartens from Buenos Aires had antibodies against Shiga-toxins. Also, it was recognized that human beings can be carriers and eliminate STEC in faeces, without presenting disease symptoms [6]. These findings allowed us to hypothesize about the role of person-to-person transmission, particularly if we consider that more than 54.0% of disease outbreaks caused by STEC worldwide were not associated with any specific food source [32,140,144].

Cooking preference impacted on the probability of HUS among Argentine consumers, but the responsibility cannot rest exclusively on consumers and their consumption habits. The origin of beef (abattoirs applying or not HACCP-STEC) was also associated with HUS risk. It would be very important to continue working in the beef production chain and to deepen the knowledge of other food production chains and sources of water supply. Additionally, person-to-person transmission should be evaluated and epidemiological studies strengthened to identify the origin of HUS cases in order to reduce the impact of HUS on the child population of Argentina.

## 5. Conclusion

In summary, the QMRA developed in the present study did not find any statistical association between primary production variables (cattle age, season and production system) and the probability of developing HUS. The model predicted almost doble *stx* prevalence and higher STEC enumeration levels in carcasses and beef cuts produced in abattoirs not applying HACCP-STEC. The abattoir type (applying or not applying HACCP-STEC), storage temperatures (higher temperatures from abattoir to home) and lack of hygienic practices at retail were the most influential factors increasing significantly HUS probability. Beef consumption in the Argentinian children population (mainly ground beef) was able to explain only about 10.0% of the HUS median cases per year in children under 15 years. This study highlights the multifactorial nature of HUS disease and the plausibility of other STEC infection routes (other food sources, animal contact, person-to-person) and the need to investigate the contribution of these additional risk factors on the overall HUS disease burden in the children population of Argentina.

## Supporting information

S1 Table Prevalence of *stx* and STEC in beef products from all over the world. (DOCX)

S2 Table Bovine slaughtered during 2018. Total amount and amount provided by feedlots.
(DOCX)

S3 Table Peer-reviewed sources of *stx* prevalence in Argentinean cattle feces used for input into the model. (DOCX)

S4 Table Scientific publications of samplings conducted in Argentinean abattoirs used to model the prevalence of *stx*-positive carcasses after slaughter. (DOCX)

S5 Table Scientific publications of samplings conducted in Argentinean retails used to model *stx* prevalence at retail. (DOCX)

S6 Table Survey of Argentinean beef consumption habits. (DOCX)
