# Supplementary material for: Quantitative risk assessment of haemolytic uremic syndrome associated with beef consumption in Argentina
Source: PLoS One. 2020 Nov 13;15(11):e0242317. doi: 10.1371/journal.pone.0242317 (PMC7665811; doi:10.1371/journal.pone.0242317)
Supplement: S1 Table — (DOCX) [file pone.0242317.s001.docx]

**S1 Table. Prevalence of *stx* and STEC in beef products from all over the world.**

| **Country** | **Foodstuff** | **N** | **Methodology** | **+ (%)** | **Reference** |
| --- | --- | --- | --- | --- | --- |
| Argentina | Carcass  Beef cut  Trimmings  Ground beef | 5879  10,405  5790  636 | Screening or  Isolation | 850 (14.4)  186 (1.8)  1882 (32.0)  176 (27.0) | S3-5 Table |
|  | Commercial hamburger | 6129 | Screening | 3746 (56.7) | Three food processing plants (industry communication) |
|  |  | 95 | Isolation | 8 (8.4) | [1] |
| Australia | Ground beef | 285 | Screening  Isolation | 74 (26.0)  45 (16.0) | [2] |
|  | Trimmings | 220 | Screening  Isolation | 67 (30.0)  9 (4.09) | [3] |
|  |  | 53 | Screening | n/r (<4.0) | [4] |
| Bangladesh | Beef cut | 60 | Screening  Isolation | 41 (68.3)  5 (8.3) | [5] |
| Brazil | Beef cut | 8 | Screening | n/r (<4.0) | [4] |
|  | Ground beef | 138 | Isolation | 0 (0.0) | [6] |
|  |  | 250 | Isolation | 4 (1.6) | [7] |
|  |  | 91 | Isolation | 2 (2.1) | [8] |
| Canada | Carcass | 1018 | Isolation | 55 (5.4) | [9] |
|  | Beef cut | 150 | Isolation | 1 (0.7) | [10] |
| Chile | Beef cut | 226 | Isolation | 2 (0.9) | [11] |
|  |  | 87 | Screening | 3 (3.4) | [4] |
|  | Ground beef | 430 | Screening  Isolation | 212 (49.3)  43 (10.0) | [12] |
| China | Beef cut | 191 | Screening  Isolation | 63 (33.0)  19 (9.9) | [13] |
|  |  | 87 | Screening | 53 (60.9) | [14] |
| Costa Rica | Beef cut | 279 | Screening | 13 (4.7) | [15] |
| Egypt | Beef cut  Ground beef  Hamburger | 27  30  30 | Isolation | 3 (11.1)  5 (16.7)  10 (33.3) | [16] |
| European Union | Carcass and beef cut | 4,603 | Screening or  Isolation | 58 (1.3) | [17] |
| France | Beef cut | 411 | Screening  Isolation | 47 (11.0)  16 (3.9) | [18] |
| Italy | Ground beef  Hamburger  Beef carpaccio | 250 | Screening  Isolation | 21 (8.4)  5 (2.0) | [19] |
|  | Beef cut | 239 | Screening | 20 (8.4) | [20] |
| India | Beef cut | 132 | Isolation | 55 (41.7) | [21] |
| Iran | Beef cut | 340 | Screening | 101 (29.7) | [22] |
|  | Ground beef | 68 | Isolation | 16 (23.5) | [23] |
| Iraq | Carcass  Ground beef  Hamburger | 120  120  120 | Isolation | 6 (23.0)  0 (0.0)  2 (28.5) | [24] |
| Ireland | Ground beef  Hamburger | 309  444 | Isolation | 4 (1.3)  5 (1.1) | [25] |
| Poland | Carcass | 406 | Isolation | 12 (3.0) | [26] |
| Nigeria | Beef cut | 448 | Isolation | 17 (3.8) | [27] |
| Spain | Ground beef | 785 | Isolation | 95 (12.0) | [28] |
| Switzerland | Ground beef | 211 | Isolation | 5 (2.3) | [29] |
| Turkey | Ground beef | 116 | Screening | 14 (12.5) | [30] |
| United Kingdom | Carcass | 450 | Screening | 122 (27.0) | [31] |
| USA | Carcass | 326 | Screening  Isolation | 43 (13.4)  33 (10.1) | [32, 33] |
|  |  | 1232 | Screening  Isolation | 200 (16.2)  125 (10.1) | [34] |
|  |  | 86 | Screening  Isolation | 20 (23.0)  6 (6.9) | [35] |
|  |  | 203 | Isolation | 8 (3.9) | [36] |
|  |  | 576 | Screening | 349 (60.6) | [37] |
|  | Beef cut | 480 | Screening  Isolation | 173 (36.0)  44 (9.1) | [35] |
|  | Ground beef | 4,133 | Screening  Isolation | 1006 (24.3)  300 (7.3) | [38] |
|  |  | 249 | Screening  Isolation | 21 (8.5)  13 (5.2) | [39] |
|  |  | 308 | Screening | 91 (29.5) | [40] |
|  | Trimmings | 487 | Screening  Isolation | 147 (30.0)  28 (5.7) | [3] |
|  |  | 3 | Screening | 0 (0.0) | [4] |
| Uruguay | Beef cut | 256 | Screening  Isolation | 72 (28.0)  40 (15.6) | [3] |
|  |  | 17 | Screening | 4 (24.0) | [4] |
| New Zealand | Trimmings | 223 | Screening  Isolation | 23 (9.7)  4 (1.8) | [3] |

N: number of samples; + (%): number and percentage of STEC-positive samples; n/r: none-reported

**References**

1. Gómez D, Miliwebsky E, Fernández Pascua C, Baschkier A, Manfredi E, Zotta M, et al. Isolation and characterization of Shiga toxin-producing *Escherichia coli* from frozen hamburgers and soft cheese. Rev Argentin Microbiol. 2002;34:6.

2. Barlow RS, Gobius KS, Desmarchelier PM. Shiga toxin-producing *Escherichia coli* in ground beef and lamb cuts: results of a one-year study. Int J Food Microbiol. 2006;111(1):5. doi: 10.1016/j.ijfoodmicro.2006.04.039. PubMed PMID: 16793157.

3. Bosilevac JM, Guerini MN, Brichta-Harhay DM, Arthur TM, Koohmaraie M. Microbiological characterization of imported and domestic boneless beef trim used for ground beef. J Food Prot. 2007;70(2):10.

4. Baeza Quiroz CB. Aislamiento y caracterización de cepas de *Escherichia coli* productor de toxina Shiga desde carne de vacuno nacional e importada, distribuída en los principales supermercados de la provincia de Santiago. Chile: Escuela de Salud Pública. Universidad Mayor; 2013.

5. Islam MA, Mondol AS, Azmi IJ, de Boer E, Beumer RR, Zwietering MH, et al. Occurrence and Characterization of Shiga Toxin–Producing *Escherichia coli* in Raw Meat, Raw Milk, and Street Vended Juices in Bangladesh. Foodborne Pathog Dis. 2010;7:6.

6. Ristori CA, Gravato Rowlands RE, Geraldes Martins C, Barbosa ML, dos Santos LF, Jakabi M, et al. Assessment of Consumer Exposure to *Salmonella* spp., *Campylobacter* spp., and Shiga Toxin–Producing *Escherichia coli* in Meat Products at Retail in the City of Sao Paulo, Brazil. Foodborne Pathog Dis. 2017;8:7.

7. Morato Bergamini AM, Simões M, Irino K, Tardelli Gomes TA, Cabilio Guth BE. Prevalence and characteristics of Shiga toxin-producing *Escherichia coli* (STEC) strains in ground beef in SãO Paulo, Brazil. Braz J Microbiol. 2007;38:5.

8. Rodolpho D, Marin JM. Isolation of Shiga toxigenic *Escherichia coli* from butcheries in Taquaritinga city, State of São Paulo, Brazil. Braz J Microbiol. 2007;38:4. doi: 10.1590/s1517-83822007000400004.

9. Bohaychuk VM, Gensler GE, Romero Barrios P. Microbiological baseline study of beef and pork carcasses from provincially inspected abattoirs in Alberta, Canada. Can Vet J. 2011;52:6.

10. Jones TH, Nattress FM, Dilts B, Olsen D, Muehlhauser V. Numbers of coliforms, *Escherichia coli*, F-RNA phage, rotavirus, bovine enteric calicivirus and presence of non-O157 STEC on commercial vacuum packaged beef. Food Microbiol. 2014;42:7. doi: 10.1016/j.fm.2014.04.001. PubMed PMID: 24929741.

11. Burgos M, C. MM, Barría B, Perez M, Ulloa M, Vaquero A, et al. Estudio de la prevalencia de *Escherichia coli* enterohemorrágica en canales de vacuno y cerdo faenadas en la región metropolitana, Chile. Av Cienc Vet. 2003;18:5.

12. Toro M, Rivera D, Jimenez MF, Diaz L, Navarrete P, Reyes-Jara A. Isolation and characterization of non-O157 Shiga toxin-producing *Escherichia coli* (STEC) isolated from retail ground beef in Santiago, Chile. Food Microbiol. 2017;75:6. doi: 10.1016/j.fm.2017.10.015. PubMed PMID: 30056963.

13. Bai X, Wang H, Xin Y, Wei R, Tang X, Zhao A, et al. Prevalence and characteristics of Shiga toxin-producing *Escherichia coli* isolated from retail raw meats in China. Int J Food Microbiol. 2015;200:8.

14. Li R, Tan X, Xiao J, Wang H, Liu Z, Zhou M, et al. Molecular screening and characterization of Shiga toxin-producing *Escherichia coli* in retail foods. Food Control. 2016;60:180-8. doi: <http://dx.doi.org/10.1016/j.foodcont.2015.07.045>.

15. Martinez-Chavez L, Cabrera-Diaz E, Perez-Montano JA, Garay-Martinez LE, Varela-Hernandez JJ, Castillo A, et al. Quantitative distribution of *Salmonella* spp. and *Escherichia coli* on beef carcasses and raw beef at retail establishments. Int J Food Microbiol. 2015;210:149-55. doi: 10.1016/j.ijfoodmicro.2015.06.016. PubMed PMID: 26125489.

16. Mohammeda AM, Sallam KI, Zaid Eldaly EA, Mohammed Ahdya A, Tamura T. Occurrence, serotypes and virulence genes of non-O157 Shiga toxin-producing *Escherichia coli* in fresh beef, ground beef, and beef burger. Food Control. 2014;37:6.

17. EFSA, ECDC. The European Union summary report on trends and sources of zoonoses, zoonotic agents and food-borne outbreaks in 2012. EFSA J. 2014;12(2):312. doi: 10.2903/j.efsa.2014.3547.

18. Pradel N, Livrelli V, De Champs C, Palcoux JB, Reynaud A, Scheutz F, et al. Prevalence and Characterization of Shiga Toxin-Producing *Escherichia coli* Isolated from Cattle, Food, and Children during a One-Year Prospective Study in France. J Clin Microbiol. 2000;38.

19. Nobili G, Franconieri I, La Bella G, Basanisi MG, La Salandra G. Prevalence of Verocytotoxigenic *Escherichia coli* strains isolated from raw beef in southern Italy. Int J Food Microbiol. 2017;257:5. doi: 10.1016/j.ijfoodmicro.2017.06.022. PubMed PMID: 28672173.

20. Varcasia BM, Tomassetti F, De Santis L, Di Giamberardino F, Lovari S, Bilei S, et al. Presence of Shiga toxin-producing *Escherichia coli* (STEC) in fresh beef marketed in 13 eegions of ITALY (2017). Microorganisms. 2018;6(126):12. PubMed PMID: 30563244; PubMed Central PMCID: PMCPMC6313577.

21. Sethulekshmi C, Latha C, Anu CJ. Occurrence and quantification of Shiga toxin-producing *Escherichia coli* from food matrices. Vet World. 11:8.

22. Momtaz H, Safarpoor Dehkordi F, Rahimi E, Ezadi H, Arab R. Incidence of Shiga toxin-producing *Escherichia coli* serogroups in ruminant's meat. Meat Sci. 2013;95(2):381-8. doi: 10.1016/j.meatsci.2013.04.051. PubMed PMID: 23747633.

23. Panahee M, Pourtaghi H. Virulence gene profiles of Shiga-Toxin producing *Escherichia coli* isolates from retail raw meat in Iran. Bul J Vet Med. 2017, 20, No 1, 19–26. 2017;20:8.

24. Taha ZM, Yassin NA. Prevalence of diarrheagenic *Escherichia coli* in animal products in Duhok province, Iraq. Iran J Vet Res. 2019;20:8.

25. FSAI. Microbiological safety of raw minced beef and beef burgers on retail sale in Ireland (11NS1). Microbiology ed. Dublin, Ireland2013. p. 25.

26. Wieczorek K, Beutin L, Osek J. Rare VTEC serotypes of potential zoonotic risk isolated from bovine hides and carcases. Vet Rec. 2011;168(3):80. doi: 10.1136/vr.c5263. PubMed PMID: 21257589.

27. Ojo OE, Ajuwape ATP, Otesile EB, Owoade AA, Oyekunle MA, Adetosoye AI. Potentially zoonotic shiga toxin-producing *Escherichia coli* serogroups in the faeces and meat of food-producing animals in Ibadan, Nigeria. Int J Food Microbiol. 2010;142:8.

28. Mora A, Blanco M, Blanco JE, Dahbi G, Lopez C, Justel P, et al. Serotypes, virulence genes and intimin types of Shiga toxin (verocytotoxin)-producing *Escherichia coli* isolates from minced beef in Lugo (Spain) from 1995 through 2003. BMC Microbiol. 2007;7:9. doi: 10.1186/1471-2180-7-13. PubMed PMID: 17331254; PubMed Central PMCID: PMCPMC1810539.

29. Fantelli K, Stephan R. Prevalence and characteristics of Shigatoxin-producing *Escherichia coli* and *Listeria monocytogenes* strains isolated from minced meat in Switzerland. Int J Food Microbiol. 2001;70:7.

30. Aslan S, Altindis M, Kara R, Demiray T, Koroglu M. Detection and evaluation of *Escherichia coli* O157:H7 and Shiga-toxin genes *stx*_1_, *stx*_2_ in cattle meat samples. Clin Lab. 2018;64:6.

31. Monaghan Á, Byrne B, Fanning S, Sweeney T, McDowell D, Bolton DJ. Serotypes and virulotypes of non-O157 shiga-toxin producing *Escherichia coli* (STEC) on bovine hides and carcasses. Food Microbiol. 2012;32:7.

32. Elder RO, Keen JE, Siragusa GR, Barkocy-Gallagher GA, Koohmaraie M, Laegreid WW. Correlation of enterohemorrhagic Escherichia coli O157 prevalence in feces, hides, and carcasses of beef cattle during processing. Proc Natl Acad Sci U S A. 2000;97(7):2999-3003. doi: 10.1073/pnas.060024897. PubMed PMID: 10725380; PubMed Central PMCID: PMCPMC16181.

33. Arthur TM, Barkocy-Gallagher GA, Rivera-Betancourt M, Koohmaraie M. Prevalence and characterization of non-O157 Shiga toxin-producing *Escherichia coli* on carcasses in commercial beef cattle processing plants. Appl Environ Microbiol. 2002;68(10):4847-52. doi: 10.1128/aem.68.10.4847-4852.2002.

34. Barkocy-Gallagher GA, Arthur TM, Rivera-Betancourt M, X. N, Shackelford SD, Wheeler TL, et al. Seasonal prevalence of Shiga toxin–producing *Escherichia coli*, including O157:H7 and non-O157 serotypes, and *Salmonella* in commercial beef processing plants. J Food Prot. 2003;66:9.

35. Cobbold RN, Davis MA, Rice DH, Szymanski M, Tarr PI, Hancock DD. Associations between bovine, human, and raw milk, and beef isolates of non-O157 Shiga toxigenic *Escherichia* coli within a restricted geographic area of the United States. J Food Prot. 2008;71(5):5.

36. Svoboda AL, Dudley EG, DebRoy C, Mills EW, Cutter CN. Presence of Shiga Toxin–Producing *Escherichia coli* O-groups in small and very-small beef-processing plants and resulting ground beef detected by a multiplex polymerase chain reaction assay. Foodborne Pathog Dis. 2013;10:7.

37. Stromberg ZR, Baumann NW, Lewis GL, Sevart NJ, Cernicchiaro N, Renter DG, et al. Prevalence of Enterohemorrhagic *Escherichia coli* O26, O45, O103, O111, O121, O145, and O157 on Hides and Preintervention Carcass Surfaces of Feedlot Cattle at Harvest. Foodborne Pathog Dis. 2015;12(7):8. doi: 10.1089/fpd.2015.1945. PubMed PMID: 26125496.

38. Bosilevac JM, Koohmaraie M. Prevalence and characterization of non-O157 shiga toxin-producing *Escherichia coli* isolates from commercial ground beef in the United States. Appl Environ Microbiol. 2011;77(6):10. doi: 10.1128/AEM.02833-10. PubMed PMID: 21257806; PubMed Central PMCID: PMCPMC3067332.

39. Ju W, Shen J, Li Y, Toro MA, Zhao S, Ayers S, et al. Non-O157 Shiga toxin-producing *Escherichia coli* in retail ground beef and pork in the Washington D.C. area. Food Microbiol. 2012;32(2):7. doi: 10.1016/j.fm.2012.07.017. PubMed PMID: 22986203.

40. Wasilenko JL, Fratamico PM, Sommers C, DeMarco DR, Varkey S, Rhoden K, et al. Detection of Shiga toxin-producing *Escherichia coli* (STEC) O157:H7, O26, O45, O103, O111, O121, and O145, and *Salmonella* in retail raw ground beef using the DuPont BAX(R) system. Front Cell Infect Microbiol. 2014;4(81):7. doi: 10.3389/fcimb.2014.00081. PubMed PMID: 24995164; PubMed Central PMCID: PMCPMC4061970.
