# Supplementary material for: Quantitative risk assessment of haemolytic uremic syndrome associated with beef consumption in Argentina
Source: PLoS One. 2020 Nov 13;15(11):e0242317. doi: 10.1371/journal.pone.0242317 (PMC7665811; doi:10.1371/journal.pone.0242317)
Supplement: S2 Table — Total amount and amount provided by feedlots. (DOCX) [file pone.0242317.s002.docx]

**S2 Table. Bovine slaughtered during 2018. Total amount and amount provided by feedlots.**

|  | **Steer** | | **Young bull** | | **Bull** | | **Cow** | | **Heifer** | | **Calf** | | **Veal** | | **All categories** | |
| --- | --- | --- | --- | --- | --- | --- | --- | --- | --- | --- | --- | --- | --- | --- | --- | --- |
| **Month** | **S-I** | **Feedlot** | **S-I** | **Feedlot** | **S-I** | **Feedlot** | **S-I** | **Feedlot** | **S-I** | **Feedlot** | **S-I** | **Feedlot** | **S-I** | **Feedlot** | **S-I** | **Feedlot** |
| Jan | 188,776 | 39,972 | 158,498 | 118,383 | 13,951 | 978 | 157,212 | 16,180 | 28,399 | 114,066 | 87,930 | 19,225 | 161,374 | 26,059 | 796,140 | 334,863 |
| Feb | 158,069 | 45,502 | 147,048 | 105,528 | 13,492 | 1,088 | 149,244 | 13,021 | 26,552 | 103,497 | 74,924 | 17,004 | 140,199 | 23,732 | 709,528 | 309,372 |
| Mar | 194,054 | 49,813 | 164,593 | 112,081 | 14,799 | 1,082 | 187,348 | 15,943 | 35,616 | 104,160 | 80,252 | 18,279 | 146,693 | 22,387 | 823,355 | 323,745 |
| Apr | 179,980 | 49,017 | 138,258 | 114,495 | 13,577 | 1,219 | 184,866 | 18,801 | 21,212 | 110,366 | 77,757 | 19,092 | 147,921 | 24,984 | 763,571 | 337,974 |
| May | 178,316 | 62,253 | 151,380 | 122,573 | 13,876 | 1,181 | 191,052 | 19,934 | 32,639 | 101,724 | 86,669 | 17,629 | 142,838 | 24,034 | 796,770 | 349,328 |
| Jun | 173,757 | 51,155 | 131,111 | 113,094 | 16,439 | 956 | 224,631 | 20,380 | 26,044 | 94,754 | 81,322 | 20,535 | 129,852 | 27,578 | 783,156 | 328,452 |
| Jul | 199,437 | 46,966 | 134,275 | 136,468 | 16,418 | 1,220 | 220,809 | 22,233 | 42,229 | 77,130 | 94,106 | 28,549 | 94,043 | 17,929 | 801,317 | 330,495 |
| Aug | 197,809 | 53,231 | 156,754 | 117,777 | 18,439 | 954 | 232,212 | 22,934 | 43,397 | 85,518 | 93,055 | 26,248 | 120,579 | 21,863 | 862,245 | 328,525 |
| Sept | 162,058 | 38,660 | 136,489 | 107,598 | 17,720 | 932 | 181,109 | 24,882 | 31,624 | 91,136 | 76,219 | 25,255 | 114,588 | 27,282 | 719,807 | 315,745 |
| Oct | 198,021 | 37,119 | 170,270 | 117,351 | 20,640 | 1,035 | 205,233 | 23,240 | 41,249 | 110,688 | 85,759 | 23,389 | 146,882 | 33,745 | 868,054 | 346,567 |
| Nov | 164,890 | 45,515 | 166,803 | 106,163 | 18,171 | 945 | 187,860 | 17,691 | 38,343 | 111,881 | 81,214 | 16,736 | 151,350 | 24,177 | 808,631 | 323,108 |
| Dec | 137,934 | 44,131 | 157,068 | 125,907 | 16,055 | 973 | 176,757 | 14,710 | 26,296 | 125,608 | 85,581 | 14,185 | 161,388 | 21,478 | 761,079 | 346,992 |
| Total | 2,133,101 | 563,334 | 1,812,367 | 1,397,418 | 193,577 | 12,563 | 2,298,333 | 229,949 | 393,600 | 1230528 | 1,004,788 | 246,126 | 1,952,955 | 295,248 | 13,468,639 | 3,975,166 |

S-I: Semi-intensive

[1]

**Reference**

1. IPCVA. [2019]. Available from: <http://www.ipcva.com.ar/>.
