# Supplementary material for: Quantitative risk assessment of haemolytic uremic syndrome associated with beef consumption in Argentina
Source: PLoS One. 2020 Nov 13;15(11):e0242317. doi: 10.1371/journal.pone.0242317 (PMC7665811; doi:10.1371/journal.pone.0242317)
Supplement: S3 Table — (DOCX) [file pone.0242317.s003.docx]

S3 Table. Peer-reviewed sources of *stx* prevalence in Argentinean cattle feces used for input into the model.

| **Season/Category/Production System** | **N** | **+** | **Reference** |
| --- | --- | --- | --- |
| **Spring-Summer/Young/Feedlot** | 31  30 | 1  6 | [1]  [2] |
| **Spring-Summer/Young/Extensive** | 108  130 | 31  114 | [2]  [3] |
| **Spring-Summer/Adult/Feedlot** | 18 | 3 | [2] |
| **Spring-Summer/Adult/Extensive** | 34  720  720  247  144 | 0  180  94  73  54 | [1]  [4]  [5]  [2]  [6] |
| **Autumn-Winter/Young/Feedlot** | 30  6  59 | 1  0  37 | [1]  [2]  [7] |
| **Autumn-Winter/Young/Extensive** | 96  70 | 12  24 | [2]  [3] |
| **Autumn-Winter/Adult/Feedlot** | 6 | 0 | [2] |
| **Autumn-Winter/Adult/Extensive** | 34  720  720  240  118  148 | 1  360  76  56  46  53 | [1]  [4]  [5]  [2]  [8]  [6] |

N= number of samples, each sample came from one animal; +: STEC-positive samples

**References**

1. Favier GI, Estrada CL, Cortinas TI, Escudero ME. Detection and Characterization of Shiga Toxin Producing *Escherichia coli*, *Salmonella* spp., and *Yersinia* Strains from Human, Animal, and Food Samples in San Luis, Argentina. Internat J Microbiol. 2014;2014:12. doi: 10.1155/2014/284649. PubMed PMID: 25177351; PubMed Central PMCID: PMCPMC4142171.

2. Masana MO, D'Astek BA, Palladino PM, Galli L, Del Castillo LL, Carbonari C, et al. Genotypic characterization of non-O157 Shiga toxin-producing *Escherichia coli* in beef abattoirs of Argentina. J Food Prot. 2011;74(12):10. doi: 10.4315/0362-028X.JFP-11-189. PubMed PMID: 22186039.

3. Meichtri L, Miliwebsky E, Gioffre A, Chinen I, Baschkier A, Chillemi G, et al. Shiga toxin-producing *Escherichia coli* in healthy young beef steers from Argentina: prevalence and virulence properties. Int J Food Microbiol. 2004;96(2):10. doi: 10.1016/j.ijfoodmicro.2004.03.018. PubMed PMID: 15364473.

4. Fernandez D, Rodriguez EM, Arroyo GH, Padola NL, Parma AE. Seasonal variation of Shiga toxin-encoding genes (*stx*) and detection of *E. coli* O157 in dairy cattle from Argentina. J Appl Microbiol. 2009;106(4):8. doi: 10.1111/j.1365-2672.2008.04088.x. PubMed PMID: 19187162.

5. Fernández D, Irino K, Sanz M, Padola NL, Parma AE. Characterization of Shiga Toxin-producing *Escherichia coli* isolated from dairy cows in Argentina. Lett Appl Microbiol. 2010;51:6.

6. Tanaro JD, Galli L, Lound LH, Leotta GA, Piaggio MC, Carbonari CC, et al. Non-O157:H7 Shiga toxin-producing *Escherichia coli* in bovine rectums and surface water streams on a beef cattle farm in Argentina. Foodborne Pathoge Dis. 2012;9(10):7. doi: 10.1089/fpd.2012.1182. PubMed PMID: 22994915.

7. Padola NL, Sanz ME, Blanco JE, Blanco M, Blanco J, Etcheverria AI, et al. Serotypes and virulence genes of bovine Shigatoxigenic *Escherichia coli* (STEC) isolated from a feedlot in Argentina. Vet Microbiol. 2004;100:7. doi: 10.1016/s0378-1135(03)00127-5.

8. Sanz ME, Viñas MR, Parma AE. Prevalence of bovine verotoxin-producing *Escherichia coli* in Argentina. Eur J Epidemiol. 1998;14:5.
