# Supplementary material for: Quantitative risk assessment of haemolytic uremic syndrome associated with beef consumption in Argentina
Source: PLoS One. 2020 Nov 13;15(11):e0242317. doi: 10.1371/journal.pone.0242317 (PMC7665811; doi:10.1371/journal.pone.0242317)
Supplement: S4 Table — (DOCX) [file pone.0242317.s004.docx]

**S4 Table. Scientific publications of samplings conducted in Argentinean abattoirs used to model the prevalence of *stx*-positive carcasses after slaughter.**

| **Applying HACCP-STEC** | **Type of sample** | **Abattoir section** | **N** | **+** | **Reference** |
| --- | --- | --- | --- | --- | --- |
| **Yes** | All carcass surface | Cold chamber | 811 | 73 | [1] |
|  | All carcass surface  Beef cut  Trimmings | Cold chamber  at final of deboning  deboning | 3205 grouped in 641  9570 grouped in 1914  3190 grouped in 638 | 37 pools  111 pools  45 pools | [2] |
|  | All carcass surface | slaughter pre-washing | 1350 | 506 | [3] |
|  | Standing animal leather  Standing animal leather  Perineum  All carcass surface | corral  stocks  post knockout  post intervention | 30  30  30  60 | 29  30  29  4 | [4] |
|  | All carcass surface  Beef cut | Cold chamber  Deboning | 165  714 | 5  58 | Brusa et al. (unpublished work) |
|  | Beef cut | n/a* | 55 | 9 | [5] |
|  | Trimmings | n/a | 2591 | 1837 | Food processing plant (industry communication) |
| **No** | All carcass surface | Cold chamber | 60 | 5 | [6] |
|  | All carcass surface  Head meat | Cold chamber  Offal area | 180  9 | 175  9 | [7] |
|  | All carcass surface | Cold chamber | 80 | 27 | [8] |
|  | Carcass (2 areas) | Cold chamber | 81 | 10 | [9] |

N= samples amount; +: samples STEC positives; n/a: not applicable; *Argentinean steam vacuum packed beef, sold at Chilean retail

**References**

1. Masana MO, D'Astek BA, Palladino PM, Galli L, Del Castillo LL, Carbonari C, et al. Genotypic characterization of non-O157 Shiga toxin-producing *Escherichia coli* in beef abattoirs of Argentina. J Food Prot. 2011;74(12):10. doi: 10.4315/0362-028X.JFP-11-189. PubMed PMID: 22186039.

2. Brusa V, Restovich V, Galli L, Teitelbaum D, Signorini M, Brasesco H, et al. Isolation and characterization of non-O157 Shiga toxin-producing *Escherichia coli* from beef carcasses, cuts and trimmings of abattoirs in Argentina. PLoS One. 2017;12(8):16. doi: 10.1371/journal.pone.0183248. PubMed PMID: 28829794; PubMed Central PMCID: PMCPMC5568767.

3. Signorini M, Costa M, Teitelbaum D, Restovich V, Brasesco H, Garcia D, et al. Evaluation of decontamination efficacy of commonly used antimicrobial interventions for beef carcasses against Shiga toxin-producing *Escherichia coli*. Meat Sci. 2018;142:8. doi: 10.1016/j.meatsci.2018.04.009. PubMed PMID: 29656275.

4. Brusa V, Restovich V, Signorini M, Pugin D, Galli L, Diaz VR, et al. Evaluation of intervention measures at different stages of the production chain in Argentinian exporting abattoirs. Food Sci Technol Int. 2019;25(6):6. doi: 10.1177/1082013219836326. PubMed PMID: 30862194.

5. Baeza Quiroz CB. Aislamiento y caracterización de cepas de *Escherichia coli* productor de toxina Shiga desde carne de vacuno nacional e importada, distribuída en los principales supermercados de la provincia de Santiago. Chile: Escuela de Salud Pública. Universidad Mayor; 2013.

6. Cap M, Carbonari CC, D'Astek BA, Zolezzi G, Deza N, Palladino MP, et al. Frequency, characterization and genotypic analysis of Shiga toxin-producing *Escherichia coli* in beef slaughterhouses of Argentina. Rev Argent Microbiol. 2019;51(1):7. doi: 10.1016/j.ram.2018.03.005. PubMed PMID: 29937134.

7. Costa M, Pracca G, Sucari A, Galli L, Ibargoyen J, Gentiluomo J, et al. Comprehensive evaluation and implementation of improvement actions in bovine abattoirs to reduce pathogens exposure. Prev Vet Med. 2020;176:8. doi: 10.1016/j.prevetmed.2020.104933. PubMed PMID: 32105862.

8. Pérez Terrazzino GB. Calidad microbiológica de la carne bovina y cuantificación del riesgo en plantas de faena de la provincia de Tucumán. Implementación de acciones de mejora. Tucumán, Argentina: Universidad Nacional de Tucumán; 2020.

9. Etcheverria AI, Padola NL, Sanz ME, Polifroni R, Kruger A, Passucci J, et al. Occurrence of Shiga toxin-producing *E. coli* (STEC) on carcasses and retail beef cuts in the marketing chain of beef in Argentina. Meat Sci. 2010;86(2):4. doi: 10.1016/j.meatsci.2010.05.027. PubMed PMID: 20646836.
