# Supplementary material for: Quantitative risk assessment of haemolytic uremic syndrome associated with beef consumption in Argentina
Source: PLoS One. 2020 Nov 13;15(11):e0242317. doi: 10.1371/journal.pone.0242317 (PMC7665811; doi:10.1371/journal.pone.0242317)
Supplement: S5 Table — (DOCX) [file pone.0242317.s005.docx]

**S5 Table. Scientific publications of samplings conducted in Argentinean retails used to model *stx* prevalence at retail.**

| **Foodstuff** | **N** | **+** | **Reference** |
| --- | --- | --- | --- |
| Beef carcass | 52 | 13 | [1] |
| Beef cut | 66 | 8 | [1] |
| Ground beef | 43  54  86  252  66  78  57 | 3  22  39  91  4  16  7 | [2]  [1]  [3]  [4]  Lopez et al. (unpublished work)  [5]  [6] |

N= number of samples; +: STEC-positive samples.

**References**

1. Etcheverria AI, Padola NL, Sanz ME, Polifroni R, Kruger A, Passucci J, et al. Occurrence of Shiga toxin-producing *E. coli* (STEC) on carcasses and retail beef cuts in the marketing chain of beef in Argentina. Meat Sci. 2010;86(2):4. doi: 10.1016/j.meatsci.2010.05.027. PubMed PMID: 20646836.

2. Barril PA, Soto SA, Jaureguiberry MV, Gottardi G, Bascur I, Leotta GA, et al. Microbiological risk characterization in butcher shops from the province of Neuquen, Patagonia Argentina. LWT - Food Sci Technol. 2019;107:6. doi: 10.1016/j.lwt.2019.02.074.

3. Leotta GA, Brusa V, Galli L, Adriani C, Linares L, Etcheverria A, et al. Comprehensive evaluation and implementation of improvement actions in butcher shops. PLoS One. 2016;11(9):16. doi: 10.1371/journal.pone.0162635. PubMed PMID: 27618439; PubMed Central PMCID: PMCPMC5019392.

4. Llorente P, Barnech L, Irino K, Rumi MV, Bentancor A. Characterization of Shiga toxin-producing *Escherichia coli* isolated from ground beef collected in different socioeconomic strata markets in Buenos Aires, Argentina. BioMed Res Int. 2014;2014:9. doi: 10.1155/2014/795104. PubMed PMID: 25006586; PubMed Central PMCID: PMCPMC4070525.

5. J. RM, Sanz M, Elichiribey L, Villalobo C, Kruger A, Colello R, et al. Carnicerías saludables: detección de *Escherichia coli* O157:H7 y no-O157:H7 en carne picada fresca y en instalaciones de comercios minoristas. XIII Congreso Argentino de Microbiología II Congreso de Microbiología Agrícola y Ambiental; Ciudad Autónoma de Buenos Aires, Argentina2013.

6. Salinas Ibáñez ÁG, Lucero Estrada C, Favier GI, Vega AE, Stagnitta PV, Mattar MA, et al. Characterization of Shiga-toxin producing *Escherichia coli* isolated from meat products sold in San Luis, Argentina. J Food Saf. 2018;38(5):1-10. doi: 10.1111/jfs.12488.
