# Supplementary material for: Quantitative risk assessment of haemolytic uremic syndrome associated with beef consumption in Argentina
Source: PLoS One. 2020 Nov 13;15(11):e0242317. doi: 10.1371/journal.pone.0242317 (PMC7665811; doi:10.1371/journal.pone.0242317)
Supplement: S6 Table — (DOCX) [file pone.0242317.s006.docx]

**S6 Table. Survey of Argentinean beef consumption habits.**

| 1. **Age** | **Responded**  **N (%)** | **Answer N (%)** | | | | | | | | | | | | | | | | | | | | |
| --- | --- | --- | --- | --- | --- | --- | --- | --- | --- | --- | --- | --- | --- | --- | --- | --- | --- | --- | --- | --- | --- | --- |
|  | 5655 (99.95) | 13 to 88 years old: 5655 (99.95) | | | | | | | | | | | | | | | | | | | | |
| **2. Do you consume beef and beef products?** | **Responded**  **N (%)** | **Yes N (%)** | | | | | | | | | | **No N (%)** | | | | | | | | | | |
|  | 5658 (100.0) | 5534 (97.8) | | | | | | | | | | 124 (2.2) | | | | | | | | | | |
| **3. How often do you consume beef products? (number of times a week you consume them)** | **Responded**  **N (%)** | **Answer N (%)** | | | | | | | | | | | | | | | | | | | | |
|  |  | **0** | **1** | | | **2** | | | **3** | | | **4** | | | **5** | | | **6** | | | | **7** |
| 3.1. Intact beef cut ^1^ | 5433 (96.0) | 121 (2.2) | 1323 (24.3) | | | 1783 (32.8) | | | 1027 (18.9) | | | 559 (10.3) | | | 367 (6.8) | | | 119 (2.2) | | | | 134 (2.5) |
| 3.2. Food made with raw ground beef ^2^ | 4856 (85.8) | 1635 (33.7) | 2326 (47.9) | | | 636 (13.2) | | | 161 (3.4) | | | 56 (1.1) | | | 22 (0.4) | | | 5 (0.1) | | | | 15 (0.3) |
| 3.3. Commercial hamburger | 4643 (82.1) | 1956 (42.1) | 2183 (47.0) | | | 362 (7.9) | | | 68 (1.5) | | | 30 (0.6) | | | 25 (0.5) | | | 4 (0.1) | | | | 15 (0.3) |
| **4. Where do you consume beef products? *** | **Responded**  **N (%)** | **Answers with multiple options* N (%)** | | | | | | | | | | | | | | | | | | | | |
|  |  | **Total** | | | | **Home** | | | | | | **Restaurant** | | | | | **Other** | | | | | |
| 4.1. Intact beef cut ^1^ | 5513 (97.4) | 6774 (100.0) | | | | 5338 (78.8) | | | | | | 1329 (19.6) | | | | | 107 (1.6) | | | | | |
| 4.2. Food made with raw ground beef ^2^ | 3909 (69.1) | 4218 (100.0) | | | | 3516 (83.4) | | | | | | 523 (12.4) | | | | | 179 (4.2) | | | | | |
| 4.3. Commercial hamburger | 3795 (97.1) | 4474 (100.0) | | | | 2551 (57.0) | | | | | | 1687 (37.7) | | | | | 236 (5.3) | | | | | |
| **5. If you consume beef products at home. Where do you buy meat?*** | **Responded**  **N (%)** | **Answers with multiple options* N (%)** | | | | | | | | | | | | | | | | | | | | |
|  |  | **Total** | | | | **Warehouse** | | | | **Butcher Shop** | | | | | **Supermarket** | | | | | **Other** | | |
| 5.1. Intact beef cut ^1^ | 5477 (96.8) | 6481 (100.0) | | | | 105 (1.6) | | | | 4478 (69.1) | | | | | 1808 (27.9) | | | | | 90 (1.4) | | |
| 5.2. Food made with raw ground beef ^2^ | 4202 (74.2) | 4650 (100.0) | | | | 75 (1.6) | | | | 3253 (70.0) | | | | | 1202 (25.8) | | | | | 120 (2.6) | | |
| 5.3. Commercial hamburger ^3^ | 3726 (65.8) | 4000 (100.0) | | | | 27 (0.7) | | | | 1069 (26.6) | | | | | 1636 (41.0) | | | | | 1268 (31.7) | | |
| **6. How is the beef product stored in the place where you buy it?*** | **Responded**  **N (%)** | **Answers with multiple options* N (%)** | | | | | | | | | | | | | | | | | | | | |
|  |  | **Total** | | | | | **Frozen** | | | | **Chilled** | | | | | | **Other** | | | | | |
| 6.1. Intact beef cut ^1^ | 5478 (96.8) | 5741 (100.0) | | | | | 531 (9.2) | | | | 5151 (89.7) | | | | | | 59 (1.1) | | | | | |
| 6.2. Food made with raw ground beef ^2^ | 3978 (70.3) | 7451 (100.0) | | | | | 485 (6.5) | | | | 3472 (46.6) | | | | | | 3494 (46.9) | | | | | |
| 6.3. Commercial hamburger | 3628 (64.1) | 3721 (100.0) | | | | | 2116 (56.9) | | | | 1346 (36.2) | | | | | | 259 (6.9) | | | | | |
| **7. How do you store beef products at home until consumption?*** | **Responded**  **N (%)** | **Answers with multiple options* N (%)** | | | | | | | | | | | | | | | | | | | | |
|  |  | **Total** | | | | | **Frozen** | | | | **Chilled** | | | | | | **Another** | | | | | |
| 7.1. Intact beef cut ^1^ | 5466 (96.6) | 6604 (100.0) | | | | | 3748 (56.7) | | | | 2832 (42.9) | | | | | | 24 (0.4) | | | | | |
| 7.2. Food made with raw ground beef ^2^ | 4074 (72.0) | 4083 (100.0) | | | | | 2033 (49.8) | | | | 1967 (48.2) | | | | | | 83 (2.0) | | | | | |
| 7.3. Commercial hamburger | 3726 (65.8) | 4016 (100.0) | | | | | 3131 (78.0) | | | | 766 (19.0) | | | | | | 119 (3.0) | | | | | |
| **8. How long does it take from buying the beef product until consumption? *** | **Responded**  **N (%)** | **Answers with multiple options* N (%)** | | | | | | | | | | | | | | | | | | | | |
|  |  | **Total** | | **0 Day** | | | **1-3 Days** | | | **3-5 Days** | | | **5-7 Days** | | | | **Weeks** | | | | **Months** | |
| 8.1. Intact beef cut ^1^ | 5443 (96.2) | 6037 (100.0) | | 994 (16.5) | | | 2697 (44.7) | | | 888 (14.7) | | | 255 (4.2) | | | | 987 (16.3) | | | | 216 (3.6) | |
| 8.2. Food made with raw ground beef ^2^ | 4066 (71.8) | 4488 (100.0) | | 1016 (22.6) | | | 1774 (39.5) | | | 589 (13.1) | | | 439 (9.8) | | | | 579 (12.9) | | | | 91 (2.1) | |
| 8.3. Commercial hamburger | 3601 (63.6) | 4003 (100.0) | | 552 (13.8) | | | 1060 (26.5) | | | 706 (17.6) | | | 519 (13.0) | | | | 949 (23.7) | | | | 217 (5.4) | |
| **9. What degree of cooking does your family prefer for beef products?** | **Responded**  **N (%)** | **Answer N (%)** | | | | | | | | | | | | | | | | | | | | |
|  |  | **Red** | | | **Medium-red** | | | | **Medium-well** | | | | | **Medium-well done** | | | | | **Well-done** | | | |
| 9.1. Intact beef cut ^1^ | 5164 (91.3) | 16 (0.3) | | | 352 (6.8) | | | | 923 (17.9) | | | | | 898 (17.4) | | | | | 2975 (57.6) | | | |
| 9.2. Food made with raw ground beef ^2^ | 4189 (74.0) | 14 (0.3) | | | 48 (1.1) | | | | 455 (10.9) | | | | | 360 (8.6) | | | | | 3312 (79.1) | | | |
| 9.3. Commercial hamburger | 3921 (69.3) | 8 (0.2) | | | 34 (0.9) | | | | 390 (9.9) | | | | | 329 (8.4) | | | | | 3160 (80.6) | | | |
| **10. How often do you accompany beef products with fresh vegetables (green leaf salads, tomatoes)?** | **Responded**  **N (%)** | **Answer N (%)** | | | | | | | | | | | | | | | | | | | | |
|  |  | **Sometimes** | | | | | | **Always** | | | | | | | | **Never** | | | | | | |
| 10.1. Intact beef cut ^1^ | 5494 (97.1) | 1761 (32.0) | | | | | | 3669 (66.8) | | | | | | | | 64 (1.2) | | | | | | |
| 10.2. Food made with raw ground beef ^2^ | 4149 (73.3) | 1781(42.9) | | | | | | 1870(45.1) | | | | | | | | 498(12.0) | | | | | | |
| 10.3. Commercial hamburger | 3858 (68.1) | 1650 (42.7) | | | | | | 1889 (49.0) | | | | | | | | 319 (8.3) | | | | | | |
| **11. Do you have two tables identified at home for the preparation of raw beef and vegetables?** | **Responded**  **N (%)** | **Answer N (%)** | | | | | | | | | | | | | | | | | | | | |
|  |  | **Yes** | | | | | | **No** | | | | | | | | **Unknown** | | | | | | |
|  | 5557 (98.2) | 2878 (51.8) | | | | | | 2609 (46.9) | | | | | | | | 70 (1.3) | | | | | | |
| **12. Do you use the tables identified for the preparation of raw beef and vegetables?** | **Responded**  **N (%)** | **Answer N (%)** | | | | | | | | | | | | | | | | | | | | |
|  |  | **Yes** | | | | | | **No** | | | | | | | | **Unknown** | | | | | | |
|  | 4876 (86.2) | 2492 (51.1) | | | | | | 2255 (46.2) | | | | | | | | 129 (2.6) | | | | | | |
| **13. Do you use the same table for raw beef and vegetables? In what order? *** | **Responded**  **N (%)** | **Answers with multiple options* N (%)** | | | | | | | | | | | | | | | | | | | | |
|  |  | **Total** | | | | | | **Meat and then vegetables** | | | | | | | | **Vegetables and then meat** | | | | | | |
|  | 3748 (66.2) | 3852 (100.0) | | | | | | 1183 (30.7) | | | | | | | | 2669 (69.3) | | | | | | |
| **14. If you use the same table to process raw beef and vegetables. Do you wash the table with detergent between these foods?** | **Responded**  **N (%)** | **Answer N (%)** | | | | | | | | | | | | | | | | | | | | |
|  |  | **Sometimes** | | | | | | **Always** | | | | | | | | **Never** | | | | | | |
|  | 4468 (79.0) | 3418 (76.5) | | | | | | 707 (15.8) | | | | | | | | 343 (7.7) | | | | | | |
| **15. After processing the raw beef. Do you wash your hands with detergent and/or soap?** | **Responded**  **N (%)** | **Answer N (%)** | | | | | | | | | | | | | | | | | | | | |
|  |  | **Sometimes** | | | | | | **Always** | | | | | | | | **Never** | | | | | | |
|  | 5493 (97.1) | 4485 (81.6) | | | | | | 887 (16.2) | | | | | | | | 121 (2.2) | | | | | | |
| **16. Do you wash the knives or other utensils used to process raw beef with detergent after use?** | **Responded**  **N (%)** | **Answer N (%)** | | | | | | | | | | | | | | | | | | | | |
|  |  | **Sometimes** | | | | | | **Always** | | | | | | | | **Never** | | | | | | |
|  | 5549 (98.1) | 5286 (95.3) | | | | | | 236 (4.2) | | | | | | | | 27 (0.5) | | | | | | |

* Answers with multiple options. Respondents were able to select more than one answer.

^1^ Anatomical beef cuts without mechanical treatments as tender.

^2^ Excepting commercial hamburgers and sausages.

^3^ The following clarification was included in the survey: “If you prepare hamburgers at home from raw minced meat, mark the option other”
